# The gut-placental axis: microbial signals remodeling maternal-fetal immune tolerance

**DOI:** 10.3389/fimmu.2026.1971075

**Published:** 2026-10-20

**Authors:** Lin Shi, Yan Li, Yanxiang Mo

**Affiliations:** Department of Obstetrics, Obstetrics and Gynecology Center, First Hospital of Jilin University, Changchun, China

**Keywords:** gut microbiota, gut-placental axis, maternal-fetal immune tolerance, preeclampsia, pregnancy complications, regulatory T cells, short-chain fatty acids, tryptophan metabolites

## Abstract

Pregnancy represents a unique immunological challenge requiring the establishment and maintenance of maternal-fetal immune tolerance. Emerging evidence demonstrates that the gut microbiota serves as a critical regulator of placental immune responses through systemic immune remodeling, constituting what is now recognized as the gut-placental immune axis. This narrative review examines the molecular pathways through which intestinal signals reach the maternal-fetal interface, encompassing short-chain fatty acids, tryptophan derivatives and their aryl hydrocarbon receptor (AhR) ligands, secondary bile acids, microbe-associated molecular patterns, bacterial extracellular vesicles, and placental exosomes. These signals collectively regulate key immune cell populations, including myeloid-derived suppressor cells, regulatory T cell subsets such as RORγt^+^ peripherally derived regulatory T cell (pTreg), Th17/Treg balance, macrophage polarization, and uterine natural killer (NK) cell function. We critically address the placental microbiome controversy, emphasizing that molecular signal transmission rather than live microbial colonization represents the prevailing paradigm for gut-placental communication. This paradigm shift redirects the research focus from identifying bacterial species to characterizing bioactive metabolites and microbe-associated molecular patterns. Furthermore, we elucidate how gut dysbiosis drives pathological cascades in major pregnancy complications, including preeclampsia, gestational diabetes mellitus, recurrent pregnancy loss, preterm birth, and fetal growth restriction. Finally, we evaluate clinical translation prospects encompassing probiotics, dietary fiber interventions, postbiotic metabolite supplementation, and engineered extracellular vesicle therapies, while acknowledging persistent challenges in establishing causal relationships, addressing individual heterogeneity, and ensuring long-term safety. This conceptual framework advances mechanistic understanding of microbiota-immune-placenta interactions and provides a foundation for developing microbiota-informed strategies to predict and prevent adverse pregnancy outcomes. Throughout, we explicitly distinguish human associative evidence from animal causal evidence.

## 1 Introduction

Pregnancy represents a unique immunological event in mammalian reproduction. The fetus expresses paternal alloantigens, yet develops to term within the maternal uterus without being rejected. Since Medawar proposed the “fetal allograft” hypothesis, this phenomenon has remained a central question in reproductive immunology (1). Traditionally, maternal-fetal immune tolerance was thought to depend primarily on local mechanisms at the maternal-fetal interface, including alterations in major histocompatibility complex expression patterns by trophoblast cells, the specialized composition of decidual immune cells, and the expansion of regulatory T cells (Tregs) (2, 3). However, mounting evidence indicates that maternal-fetal immune tolerance is not an isolated event within the uterus, but is subject to systemic regulation by the maternal immune state. Among these regulators, the gut microbiome, as the host’s largest reservoir of commensal microorganisms, remotely shapes the placental immune microenvironment through metabolic and immune signals, constituting the so-called “gut-placental immune axis” (4, 5).

The gut microbiome plays a foundational role in the development and homeostasis of host immunity. Germ-free (GF) animal studies have demonstrated that the absence of commensal microbiota leads to underdeveloped gut-associated lymphoid tissue, imbalanced T cell subset differentiation, and defective systemic immune responses (6, 7). During pregnancy, the maternal gut microbiota undergoes significant remodeling, and its metabolites distribute throughout the body via the portal vein and systemic circulation. These metabolites can cross the placental barrier or act directly on placental cells, thereby coupling maternal intestinal ecology with the fetal developmental environment in both space and time (8, 9). A recent Cell study directly confirmed the existence of this axis: gut microbiota-dependent tryptophan derivatives activated myeloid-derived suppressor cells (MDSCs) and intestinal retinoic acid receptor-related orphan receptor γt (RORγt)^+^ Tregs, suppressing excessive interferon-γ (IFN-γ) and interleukin (IL)-17 responses at the maternal-fetal interface. GF mice exhibited dysregulated T cell responses at the maternal-fetal interface and high rates of fetal resorption; supplementation with indole-3-carbinol or tryptophan-metabolizing *Lactobacillus murinus* partially rescued this phenotype. The study also observed dysregulation of these cells and metabolites in human recurrent pregnancy loss (RPL) cases (10). This work connected gut microbiota, systemic immunity, and placental immune tolerance into a complete causal chain, marking the field’s transition from correlative description to mechanistic analysis.

Beyond physiological tolerance, imbalance of the gut-placental axis is closely associated with multiple pregnancy complications. In preeclampsia (PE), gestational diabetes mellitus (GDM), RPL, preterm birth (PTB), and fetal growth restriction (FGR), maternal gut dysbiosis, altered microbiota metabolite profiles, and placental inflammation or immune tolerance defects have been consistently observed (11–13). Early prediction models and intervention strategies based on microbiota have emerged, making this axis a hotspot for translational research in perinatal medicine (14, 15).

From the perspective of signal flow, the gut’s shaping of placental immunity can be summarized along three main lines. The first is the metabolite line: soluble small molecules such as short-chain fatty acid (SCFA)s, tryptophan derivatives, and secondary bile acids reach the maternal-fetal interface via the portal vein and systemic circulation, regulating immune cell fate through receptor and epigenetic mechanisms (10, 16). The second is the microbe-associated molecular pattern (MAMP)s line: when microbiota dysbiosis occurs, molecules such as lipopolysaccharide (LPS) penetrate the intestinal barrier and trigger placental inflammatory cascades via pattern recognition receptors such as Toll-like receptor (TLR)s (17). The third is the vesicle line: bacterial extracellular vesicles and placental exosomes transmit bioactive molecules in a “packaged and concentrated” manner, enabling bidirectional long-distance communication (18, 19). These three lines are not isolated; metabolites can alter the response threshold of immune cells to MAMPs, while vesicles can simultaneously carry metabolites and nucleic acid signals, together constituting a multi-layered regulatory network (4) (**Figure 1**). Across all three lines, a unifying principle is that the effect of a given signal is dose- and context-dependent: the same molecule can maintain tolerance at physiologic, barrier-gated exposure and drive inflammation when exposure exceeds threshold or the barrier fails, and the direction of its effect can change across gestational phases.

**Figure 1:**
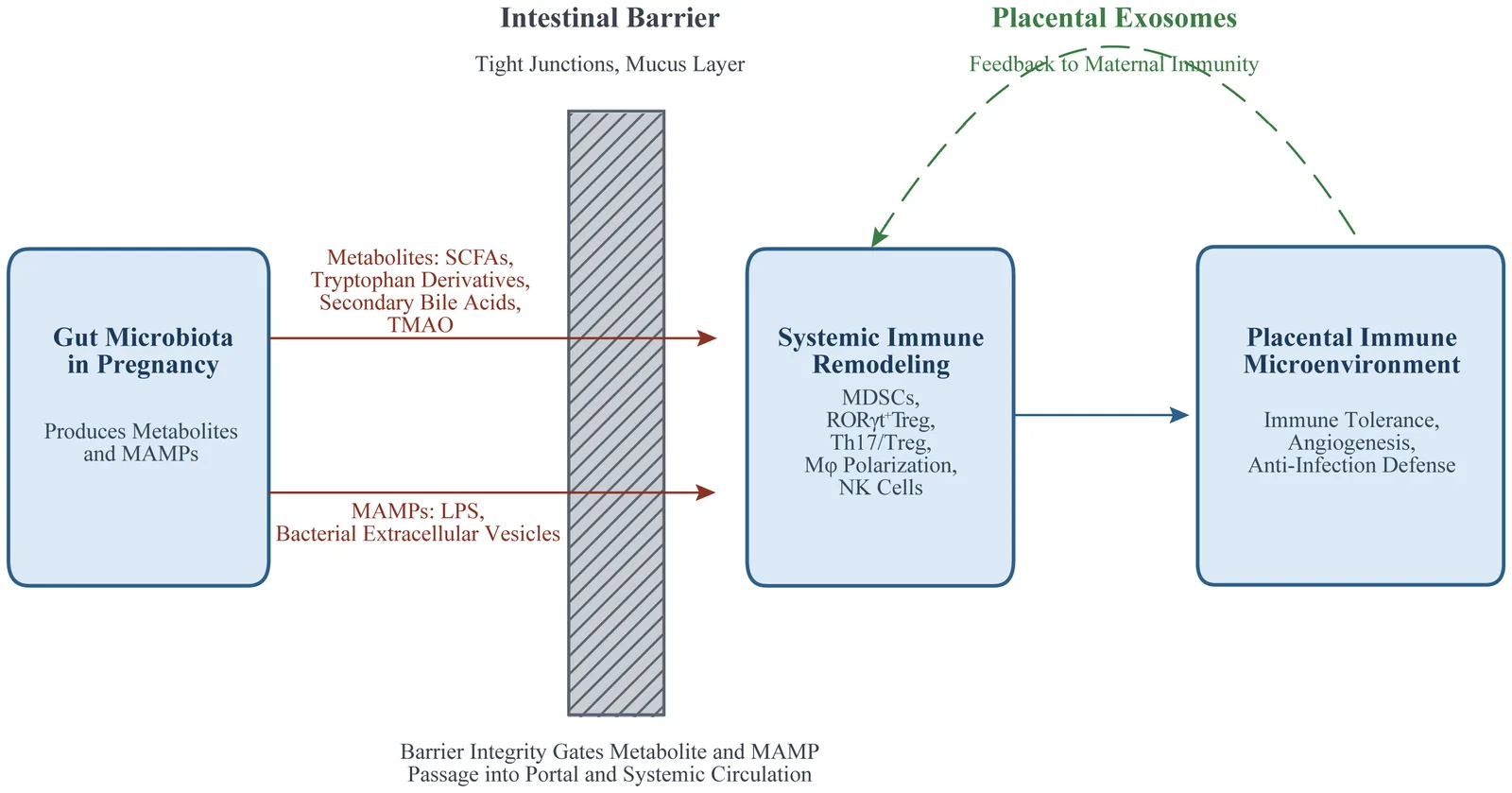
Overview of the gut, systemic immunity, and placenta axis. Pregnancy-remodeled gut microbiota produce metabolites and MAMPs that cross the intestinal barrier into the portal and systemic circulation. The barrier, composed of tight junctions and the mucus layer, gates this passage. Metabolites include SCFAs, tryptophan derivatives, secondary bile acids, and trimethylamine N-oxide (TMAO); MAMPs include LPS and bacterial extracellular vesicles. These signals drive systemic immune remodeling through modulation of MDSCs, RORγt^+^ Tregs, the Th17/Treg balance, macrophage polarization, and natural killer (NK) cells. The remodeled immune state shapes the placental immune microenvironment, including immune tolerance, angiogenesis, and anti-infection defense. Placental exosomes are released into the maternal circulation and provide feedback to maternal immunity, establishing a bidirectional, coordinated axis.

From an evolutionary perspective, the immunological challenge faced by placental mammals is more severe than that of marsupials and oviparous animals: direct contact between chorionic villi and maternal blood sinuses exposes fetal antigens continuously to the maternal immune system. To address this challenge, placental mammals have evolved multi-layered tolerance mechanisms, including the non-classical expression of trophoblast antigen presentation molecules, tissue-specific differentiation of decidual immune cells, and the emergence of pregnancy-specific regulatory cell subsets (1, 20). Concurrently, the co-evolution of commensal microbiota with the host has deeply embedded the gut microbiome into the host immune calibration system, so that microbiota remodeling during pregnancy is endowed with immunological significance beyond digestion and metabolism (3, 9).

From a historical perspective, the field has evolved through three stages. The first stage focused on local mechanisms at the maternal-fetal interface, establishing the core roles of Tregs, uNK cells, and trophoblast human leukocyte antigen (HLA) expression (21, 22). The second stage was marked by the rise of microbiomics and comprehensive characterization of microbiota remodeling during pregnancy, though the placental microbiome controversy exposed methodological risks in low-biomass research (8, 23). The third stage, the current one, is characterized by causal mechanistic analysis, with combinations of fecal microbiota transplantation, gnotobiotic animals, and single-cell technologies progressively connecting the “microbiota-metabolism-immunity-placenta” chain (10, 12).

This comprehensive narrative review elaborates on the main theme of “how intestinal signals and systemic immunity shape placental immune responses.” It first outlines the basic characteristics of the immune microenvironment at the maternal-fetal interface and the remodeling patterns of maternal gut microbiota during pregnancy. It then focuses on the molecular pathways of intestinal signal transmission to the placenta, the cellular basis of systemic immune remodeling, and the remodeling outcomes of the placental immune microenvironment. Using PE as a representative example, it analyzes axis imbalance mechanisms under pathological conditions, and finally discusses clinical translation prospects and existing challenges.

Relationship to recent reviews. Barrientos et al. (4) recently proposed that the gut–placental relationship is best understood as molecular signal transmission rather than microbial colonization, a framing this review shares and explicitly builds upon. The present review extends that framework in three ways: first, it integrates the most recent causal evidence (2023–2026), including the Cell study establishing the microbiota–MDSC/RORγt^+^ Treg–maternal-fetal tolerance axis (10), the Gut prospective cohort demonstrating pre-diagnostic microbiota-driven inflammation in GDM (12), and the Circulation Research fecal transplantation study in PE (11); second, it explicitly grades human versus animal evidence throughout the complication and translation sections; and third, it develops the dose- and context-dependence of microbial signals as an organizing principle, with implications for intervention windows.

Literature search and selection. We searched PubMed and Web of Science for articles published from January 2000 to June 2026 using combinations of the terms “gut microbiota”, “microbiome”, “placenta”, “maternal-fetal interface”, “preeclampsia”, “gestational diabetes”, “recurrent pregnancy loss”, “preterm birth”, “fetal growth restriction”, “short-chain fatty acids”, “tryptophan”, “bile acids”, “Treg”, and “MDSC”. We prioritized mechanistic studies in humans and in animal models (germ-free mice, fecal microbiota transplantation models, and pigs) and integrated recent systematic reviews and meta-analyses; articles were selected for their direct relevance to microbial signal transmission, systemic immune remodeling, or placental immune outcomes. This is a narrative rather than a systematic review, and the description above is provided to ensure transparency of sources.

## 2 The immune microenvironment at the maternal-fetal interface

### 2.1 Theoretical framework of maternal-fetal immune tolerance

The maternal-fetal interface is composed of maternal decidua and fetal-derived placental villi, representing the site of direct contact between tissues of two different genetic backgrounds. Pregnancy is not a state of traditional immunosuppression, but rather a finely regulated immune balance: the maternal immune system must tolerate the semi-allogeneic fetus while maintaining defense against pathogens (3). Mor et al. proposed that pregnancy can be divided into three immunological phases: a pro-inflammatory phase around implantation, an anti-inflammatory phase during mid-pregnancy, and a return to pro-inflammatory status before delivery, suggesting that placental immune responses possess dynamic plasticity (3). This phase framework is significant for understanding the meaning of microbiota signals: the same microbial molecule may produce different effects in different phases. Moderate inflammation during the peri-implantation period is beneficial for decidualization and vascular remodeling, whereas sustained inflammation during mid-pregnancy disrupts tolerance homeostasis.

The cellular basis of maternal-fetal immune tolerance has been established in multiple classical studies. Aluvihare et al. demonstrated in 2004 that CD4^+^CD25^+^ Tregs mediate maternal tolerance to fetal alloantigens, and that removal of Tregs significantly increases the resorption rate of allogeneic pregnancies (21). Samstein et al. further discovered that placental mammals evolved mechanisms for extrathymic induction of Tregs (20). Rowe et al. confirmed that pregnancy induces memory Tregs specific to fetal antigens, which provide rapid tolerance responses upon re-pregnancy (24). On the fetal side, Mold et al. found abundant functional, maternal antigen-specific Tregs in fetal lymph nodes by mid-pregnancy, indicating that fetal tolerance to maternal antigens is active and bidirectional rather than a state of immune blankness (25) Beyond adaptive immunity, Munn et al. reported in 1998 that indoleamine 2,3-dioxygenase (IDO) prevents allogeneic fetal rejection by degrading tryptophan and inhibiting maternal T cell activation (26). Subsequent studies further revealed that IDO expression in the decidual microenvironment is regulated by both hormonal and inflammatory signals, and that imbalance in its activity is associated with unexplained miscarriage (27).

### 2.2 Immune cell composition of the decidua and placenta

The immune cell composition at the maternal-fetal interface differs significantly from that in the periphery. In early pregnancy decidua, uterine natural killer (uNK) cells account for approximately 70% of decidual lymphocytes, equivalent to roughly 30%–40% of total decidual leukocytes. Their phenotype is predominantly CD56^bright^CD16^-^, with weak cytotoxicity but strong cytokine secretion capacity. Through secretion of angiogenic factors and chemokines, they participate in the regulation of spiral artery remodeling and trophoblast invasion (28, 29). Single-cell transcriptomics and organoid co-culture studies have shown that cytokine signals from uNK cells can promote extravillous trophoblast (EVT) differentiation along invasive pathways and influence blood flow, nutrient transport, and immune-related transcriptional programs; their functional dysregulation is associated with PE and other diseases (30). uNK cells recognize HLA-C expressed by EVT through killer cell immunoglobulin-like receptor (KIR) on their surface. The combination of maternal KIR and fetal HLA-C genotypes determines the depth of trophoblast invasion, and imbalanced combinations are associated with PE and recurrent miscarriage risk (22). The combination of maternal KIR AA haplotype with fetal HLA-C2 results in excessive inhibition of uNK cells, insufficient trophoblast invasion, and shallow spiral artery remodeling, forming the genetic susceptibility basis for PE and FGR. Conversely, activating KIR combinations are associated with better placentation (22, 31).

Decidual macrophages (dMφ) account for approximately 20%–30% of decidual leukocytes. In normal pregnancy, they predominantly exhibit an immunoregulatory (M2-like) phenotype, participating in apoptotic cell clearance, tissue remodeling, and immune tolerance maintenance (32). It should be noted that the M1/M2 dichotomy is a simplification of a continuous polarization spectrum: early pregnancy dMφs can simultaneously express classical activation and alternative activation markers, and their subset classification depends on high-resolution characterization by single-cell technologies (32). The human placenta can shape dMφ phenotype through locally secreted signals, conferring characteristics of both antigen presentation and tolerance induction (33). Fetal-derived macrophages within placental villi, known as Hofbauer cells (HBC), originate from yolk sac-derived macrophage progenitors and appear in early pregnancy, with pro-angiogenic and tissue repair functions. Single-cell studies have shown that their transcriptional characteristics evolve dynamically during pregnancy and share partial transcriptional programs and response patterns with fetal brain microglia (34–36). HBC and dMφ belong to the fetal and maternal sides respectively, and can produce different responses to the same inflammatory stimulus. This compartmentalized difference is key to understanding placental immune outcomes (35).

Regarding T cells, the decidua contains highly activated Treg subsets. Single-cell transcriptomics combined with T cell receptor sequencing of human early pregnancy decidua and peripheral blood CD4^+^ T cells identified a subset of C-C motif chemokine receptor 8 (CCR8)^+^ decidual Tregs with strong immunosuppressive function. This subset is significantly reduced in RPL patients and in a miscarriage-prone mouse model; adoptive transfer of CCR8^+^ Tregs can rescue fetal loss in the miscarriage-prone mouse model (37). This study also found that the ligand for CCR8, CCL1, is mainly produced by decidual myeloid cells, suggesting that decidual myeloid cells recruit and maintain functional Treg subsets through the chemokine axis, constituting a tolerance circuit at the cellular interaction level. Additionally, regulatory KIR^+^CD8^+^ T cells increase in human peripheral blood during mid-pregnancy and can suppress maternal T cell alloresponses against fetal antigens (38). This study also observed higher frequencies of KIR^+^CD8^+^ T cells in pregnancies with male fetuses, and that these cells could specifically suppress maternal CD8^+^ T cells targeting male-specific antigens, suggesting that this subset performs precise tolerance functions against fetal gender-related antigens.

Beyond immune cells, decidual stromal cells and glandular epithelial cells provide tissue niches for the above immune cells through secretion of colony-stimulating factors, chemokines, and extracellular matrix components. Single-cell resolution studies have charted the cellular anatomical atlas of the human maternal-fetal interface, identifying ligand-receptor interaction networks between decidual stromal cells, pericytes, and various immune cell subsets, providing a reference for understanding the cellular targets of microbiota signals at the maternal-fetal interface (39). The composition of decidual immune cells evolves dynamically with gestational stage: early pregnancy is dominated by uNK cells and macrophages, T cell proportions rise in mid to late pregnancy, and inflammatory signals resurge at term to initiate labor. This temporal characteristic means that the immunological consequences of microbiota signals depend on the gestational stage at which exposure occurs (3, 39).

### 2.3 Immunological characteristics of trophoblast cells

Trophoblast cells constitute the fetal-side barrier of the maternal-fetal interface. Syncytiotrophoblasts do not express classical HLA class I and II molecules, while EVTs express non-classical molecules such as HLA-C, HLA-E, and HLA-G, thereby escaping classical recognition by maternal CD8^+^ T cells while forming specific dialogues with uNK cells and T cells through KIR and inhibitory receptors (40). HLA-G binds to inhibitory receptors ILT2, ILT4, and KIR2DL4, implementing dual regulation of trophoblast invasion depth and decidual immune cell activation; reduced expression is associated with RPL risk (41). As a syncytial structure directly bathed in maternal blood, the syncytiotrophoblast possesses immunological characteristics combining barrier, secretory, and antigen presentation-like functions. The process of syncytialization itself is accompanied by reconstruction of the immunomodulatory molecule expression profile (40). The glycocalyx covering the syncytiotrophoblast surface and syncytial membrane vesicles constitute the first physical barrier. Secreted hCG, progesterone, and pregnancy-specific glycoproteins systemically regulate maternal immune cell behavior. Microvillous fragments shed during syncytiotrophoblast renewal enter maternal circulation, forming the physiological basis for continuous maternal “sampling” of placental materials (40, 42).

Trophoblast cells express various pattern recognition receptors, including TLRs and NOD-like receptors, which can recognize MAMPs and initiate downstream inflammatory or tolerance programs. The intensity of their responses is closely related to pregnancy outcomes (17). Different TLRs show compartmentalized distribution in trophoblasts: TLR2 and TLR4 mainly recognize Gram-positive bacterial peptidoglycan and Gram-negative bacterial LPS, while TLR3, TLR7, and TLR8 recognize viral nucleic acids. Their downstream signaling through nuclear factor kappa B (NF-κB) and interferon regulatory factors determines inflammatory or antiviral outcomes (43). Another characteristic of trophoblast PRR responses is that ligand dose and exposure duration determine outcomes: low-dose, pulsed exposure can induce antimicrobial peptide and tolerance molecule expression, whereas sustained high-dose exposure triggers pro-inflammatory cascades and apoptosis. This dose-response relationship provides a framework for understanding the distinction between physiological microbiota signals and pathological endotoxemia (17). Trophoblast cells also continuously release extracellular vesicles, delivering placenta-derived proteins, lipids, and nucleic acids into maternal circulation, constituting an important carrier for placental information transmission to the maternal systemic immune system (19).

## 3 Remodeling of maternal gut microbiota during pregnancy

Accompanied by characteristic changes in maternal metabolism, endocrinology, and immune status, the gut microbiota also undergoes dynamic remodeling during pregnancy. Koren et al.’s prospective analysis of maternal fecal microbiota in early and late pregnancy found that as pregnancy progresses, microbiota alpha diversity decreases, inter-individual beta diversity increases, and pro-inflammatory associated taxa such as Proteobacteria and Enterobacteriaceae are enriched. Transplantation of late pregnancy maternal microbiota into GF mice can induce insulin resistance and fat accumulation, suggesting that microbiota remodeling is an active component of maternal metabolic adaptation rather than a passive consequence (8). This study pioneeringly defined “pregnancy microbiota” as a biological variable with functional consequences, and its fecal microbiota transplantation strategy to verify causality has been adopted by similar studies for over a decade thereafter. Nuriel-Ohayon et al. found that elevated progesterone levels in late pregnancy can selectively promote the relative abundance of genera such as Bifidobacterium, and exogenous progesterone treatment can mimic this effect, suggesting that hormones are upstream signals driving microbiota remodeling (44). The enrichment of Bifidobacterium has potential adaptive significance: this genus is the dominant bacterium in early infant gut colonization, and maternal enrichment during pregnancy may reserve species for vertical transmission, reflecting the biological logic of “preparing for delivery and lactation.” Studies based on larger cohorts further indicate that maternal gut microbiota remodeling patterns are influenced by parity, with differences in microbiota trajectories between primiparous and multiparous women, suggesting that reproductive history participates in setting the microbiota baseline (45). Beyond bacteria, the gut mycobiome also shows dynamic changes during pregnancy and is associated with host metabolic indicators, suggesting that multi-kingdom microbial interactions participate in pregnancy adaptation (46).

Intestinal barrier function also changes during pregnancy. To meet fetal nutritional demands, maternal intestinal length and absorption area increase, while intestinal permeability rises during specific pregnancy stages, providing a structural basis for the systemic dissemination of microbiota-derived molecules (47). The intestinal barrier is composed of mechanical barriers (tight junction proteins such as claudins and zonula occludens-1), the mucus layer, immune barriers (secretory IgA and antimicrobial peptides), and the microbiota barrier. Under normal conditions, the intestinal mucosal barrier and mucus layer restrict microbial components from entering the bloodstream. When microbiota dysbiosis, high-fat diet, or inflammation damages barrier integrity, LPS and other MAMPs translocate in increased amounts, forming low-grade metabolic endotoxemia and constituting the pathological basis for intestinal signals to remotely influence the placenta (48). In a GDM animal model, highly fermentable dietary fiber precisely alleviated placental inflammation by repairing the intestinal barrier and blocking LPS translocation, corroborating from the opposite side the gating role of intestinal barrier integrity for this axis (48).

Maternal metabolic adaptation and microbiota remodeling during pregnancy are mutually causal. To meet fetal glucose and amino acid demands, the mother develops physiological insulin resistance in mid to late pregnancy, and Koren et al.’s fecal microbiota transplantation experiment proved that the microbiota itself is sufficient to drive this metabolic shift (8). SCFAs produced by microbiota fermentation of dietary fiber promote glucagon-like peptide 1 and peptide YY secretion through enteroendocrine cells, participating in the fine regulation of glucose and lipid metabolism during pregnancy. When fermentation substrates are insufficient, the microbiota turns to utilizing host mucus glycans, potentially weakening the mucus layer and promoting barrier permeability (16, 47). The above metabolism-barrier coupling suggests that dietary structure during pregnancy is the primary controllable variable determining the “quality” and “quantity” of intestinal signals.

## 4 The placental and intrauterine “microbiome” controversy: lessons from low-biomass research

Discussing the influence of intestinal signals on the placenta requires first clarifying a prerequisite question: whether the normal placenta and intrauterine environment possess an inherent microbiome. Aagaard et al. reported in 2014 that the human placenta harbors a unique microbiome, with composition similar to oral microbiota, and suggested its association with adverse pregnancy outcomes (49). Collado et al. subsequently reported detectable microbial signals in placenta and amniotic fluid, proposing that intrauterine colonization may begin within the uterus (50). The above “intrauterine microbiome” hypothesis once attracted widespread attention.

However, this hypothesis soon faced systematic methodological challenges. Perez-Muñoz et al.’s critical assessment of evidence for “sterile womb” versus “intrauterine colonization” pointed out that amplicon sequencing of low-biomass samples is highly susceptible to reagent and environmental contamination, and that early positive results could not exclude contamination sources (51). de Goffau et al. conducted multimodal detection on over 500 placental samples and found that, except for potential pathogens such as Group B Streptococcus, the placenta does not possess a reproducibly detectable inherent microbiome. Previously reported signals could be explained by batch effects and contamination (23). Multiple independent studies reached consistent conclusions: there is no evidence for a placental microbiome in term human placenta (52); meconium before vaginal delivery does not possess a detectable microbial community (53); after removing “kitome” contamination, unique placental microbiota signals could not be detected (54). In low-biomass samples, the number of bacterial DNA molecules per microliter of extract can be as low as single digits, and background signals introduced by reagents, consumables, and even sequencing batches are sufficient to overwhelm true signals, representing the common fatal weakness of early positive studies (55, 56). Kennedy et al. utilized strict contamination control procedures to prove that fetal meconium does not possess a detectable microbiome before birth (57). A 2023 Nature commentary used the “fetal microbiome” controversy as an example to illustrate the pitfalls of low-biomass microbial research, emphasizing the necessity of negative controls and multi-center validation (55). Systematic reviews also noted that the quality of evidence supporting a healthy pregnancy placental microbiome is generally low (58).

Advances in contamination control technology were key to clarifying the controversy. Davis et al. developed the *decontam* statistical method, which can identify and remove contaminant sequences in marker gene and metagenomic data based on DNA concentration gradients, becoming a standard tool for low-biomass research (56). Studies targeting early pregnancy placenta still could not obtain robust evidence supporting inherent placental microbiota existence after strict contamination control (52). It should be emphasized that negating an inherent microbiota does not equate to negating intrauterine microbial exposure: in ascending intrauterine infections, pathogens can clearly invade the amniotic cavity and endanger pregnancy. “Sterile” only refers to the absence of commensal colonization under healthy steady-state conditions.

This controversy has important methodological significance for the theme of this chapter. First, the “gut-placental axis” should not be understood as intestinal bacteria directly colonizing the placenta, but rather as microbiota-derived molecular signals (metabolites, MAMPs, extracellular vesicles) acting on the placenta through circulation. Second, bacterial metabolites of maternal origin, rather than live bacteria themselves, can be detected in fetal intestinal contents, indicating that transplacental transmission is primarily of soluble molecules (59). This study conducted metabolomic analysis of small intestinal contents from fetuses in mid-trimester pregnancy terminations, identifying multiple microbiota-derived metabolites, including tryptophan derivatives and short-chain fatty acid-related molecules, providing direct human evidence for the “molecular transplacental transmission” paradigm. Third, negative results also have scientific value: the consensus that the placenta lacks an inherent microbiome has shifted research focus from “what bacteria are in the placenta” to “which molecules reach the placenta”, a question transformation that directly spawned the current metabolite-oriented research strategy (4, 55). Kimura et al. confirmed that maternal microbiota-derived SCFAs can influence embryonic metabolic phenotypes via the placenta, and that maternal microbiota absence during pregnancy increases offspring susceptibility to metabolic disorders in adulthood (60). Gomez de Agüero et al. proved that maternal microbiota during pregnancy can drive early postnatal innate immune development in offspring through antibodies and metabolites, a process that does not require live bacteria to enter the fetal body (61). The key to this study lies in the transient colonization of GF dams only during pregnancy, with the microbiota cleared before delivery. No colonizing bacteria were detected in offspring intestines, yet lamina propria immune cell numbers and antimicrobial peptide expression were significantly elevated, thereby strictly attributing the effect to microbiota molecular signals transmitted during pregnancy. Therefore, “molecular signal transmission rather than microbial colonization” has become the mainstream paradigm in this field. It should nonetheless be acknowledged that a minority of studies, using highly sensitive or culture-based approaches, continue to report low-abundance microbial signals in the placenta; however, the weight of analyses with rigorous contamination controls does not support the existence of a stable, reproducible placental microbiome in healthy pregnancies (51, 55, 58).

## 5 Molecular pathways of intestinal signal transmission to the placenta

### 5.1 Short-chain fatty acids

SCFAs are firmly established as the main metabolites produced by intestinal anaerobic bacterial fermentation of dietary fiber, including acetate, propionate, and butyrate. SCFA concentrations in the colonic lumen can reach tens of millimoles per liter, entering the liver via the portal vein and partially entering systemic circulation. The immunomodulatory functions of SCFAs were established in three classical studies in 2013: Furusawa et al. proved that commensal bacteria-derived butyrate induces colonic Treg differentiation (mouse; *in vitro*) (62); Arpaia et al. reported that commensal metabolites promote peripheral Treg generation (mouse; *in vitro*) (63); Smith et al. confirmed that SCFAs regulate colonic Treg homeostasis through G protein-coupled receptors and epigenetic mechanisms (mouse; *in vitro*) (64). Butyrate inhibits histone deacetylase (HDAC), enhancing histone acetylation at the forkhead box P3 (Foxp3) gene locus, thereby stabilizing the Treg phenotype. Concurrently, SCFAs regulate colonic epithelial and lamina propria immune cells through receptors such as G protein-coupled receptor (GPR) 43 and GPR109A (16).

The receptor and signaling mechanisms of SCFAs are now relatively clear. GPR43 (free fatty acid receptor 2) and GPR109A mediate SCFA regulation of colonic epithelial barrier and lamina propria immune cells, while GPR41 (free fatty acid receptor 3) is expressed in sympathetic nerves and placental tissues. Kimura et al. proved that maternal microbiota-derived SCFAs during pregnancy shape offspring metabolic system development through embryonic GPR41 and GPR43 signaling (germ-free dams and GPR41/GPR43-knockout mice), with offspring of GF dams showing significantly increased susceptibility to adult obesity (60). At the epigenetic level, butyrate enhances histone acetylation at the Foxp3 promoter and conserved non-coding sequence regions through HDAC inhibition, stabilizing the Treg lineage (62, 64). Simultaneously, butyrate promotes intestinal epithelial tight junction protein expression and inhibits pro-inflammatory macrophages, forming a dual “barrier-immunity” protection (16).

In the pregnancy context, the role of SCFAs expands from local intestinal effects to the maternal-fetal interface. A 2023 Science Advances study showed that maternal microbiota supports mouse placental development: clearance of maternal gut microbiota leads to placental growth restriction and impaired fetal-placental vascularization, while SCFAs can promote tube formation in cultured vascular endothelial cells and prevent placental growth restriction and vascular insufficiency in undernutrition models (mouse; *in vitro*) (65). Another 2023 study in a GDM model found that highly fermentable dietary fiber acts through the “gut microbiota-SCFA-placental inflammation” axis: dietary fiber increases Lachnospiraceae abundance and butyrate levels, reducing placental tumor necrosis factor-α (TNF-α)-related inflammation by enhancing intestinal barrier and inhibiting LPS translocation, ultimately improving insulin resistance and pregnancy outcomes (rat GDM model) (48). The role of SCFAs in fetal development and metabolic programming has been reviewed in depth (66). The placental transport and receptor mechanisms of SCFAs are also gradually being clarified: the placenta expresses monocarboxylate transporters and free fatty acid receptors, and maternal circulating SCFAs can enter fetal circulation via syncytiotrophoblast and act on embryonic tissues (60). Maternal microbiota clearance experiments showed synchronous decreases in SCFA levels in maternal plasma and fetal mice, confirming the microbiota dependency of fetal SCFA exposure. Absence of embryonic SCFA sensing can continuously affect offspring adult metabolic phenotypes, reflecting the time window characteristics of developmental programming (60). Studies in pigs, a large animal model whose placental structure and pregnancy physiology closely resemble those of humans, have demonstrated that SCFAs regulate fetal intestinal permeability, motility, and gene expression, supporting the use of this model to investigate the perinatal effects of microbial metabolites (67). At the clinical level, GDM patients show characteristic changes in maternal circulating SCFA profiles (human association) (68). Animal experiments show that sodium butyrate supplementation can alleviate rat PE-like phenotypes by improving gut microbiota and barrier function, including decreased blood pressure, reduced proteinuria, and decreased placental inflammatory factors (69). The direct protective effect of SCFAs on placental vascular endothelium has also been verified *in vitro* models, suggesting that their effects are independent of systemic metabolic changes (*in vitro*) (65) (**Table 1**; **Figure 2**). In sum, SCFAs illustrate the central principle of this axis: physiologic, barrier-gated exposure favors tolerance and angiogenesis, whereas barrier failure couples the same metabolite line to LPS-driven inflammation.

**Table 1:** Intestinal signals in systemic and placental immunity.

Signal type	Representative molecules and sources	Systemic immune effects	Placental immune outcomes	References
SCFAs	Acetate, propionate, butyrate; produced by microbiota fermentation of dietary fiber	HDAC inhibition, promotion of Treg differentiation and M2-like macrophage polarization, enhancement of intestinal barrier	Promotion of placental vascularization, inhibition of placental inflammation, enhancement of immune tolerance	(48, 64, 65)
Tryptophan metabolites	Indole-3-carbinol, kynurenine; microbial indole pathway and host IDO pathway	MDSC activation, recruitment of RORγt^+^ pTreg, AhR activation	Suppression of IFN-γ^+^ and IL-17^+^ T cells at maternal-fetal interface, tolerance establishment	(10, 26)
Secondary bile acids	3-oxolithocholic acid, isolithocholic acid; converted by Bacteroides and other microbiota	Inhibition of Th17 differentiation, maintenance of RORγt^+^ Treg homeostasis	Potential tolerance regulation (direct evidence in pregnancy awaits establishment)	(70, 71)
MAMPs (during microbiota dysbiosis)	Gram-negative bacterial LPS, etc.; translocate into blood after intestinal barrier damage	TLR4/NF-κB activation, Th17/Treg imbalance, macrophage M1 polarization	Placental inflammation, trophoblast damage, mitochondrial dysfunction	(17, 72)
TMAO	Generated by microbiota-host co-metabolism of choline/L-carnitine	Pro-inflammatory, vascular endothelial activation	Elevated PE risk (effects are context-dependent)	(73, 74)
Placental exosomes	Derived from syncytiotrophoblast, carrying miRNAs and proteins	Circulating monocyte reprogramming, Treg expansion	Systemic immune tolerance amplification; pathological states carry sFlt-1 and other pathogenic factors	(75, 76)
Bacterial extracellular vesicles	Derived from Gram-negative bacteria and oral pathogens	Concentrated delivery of MAMPs, remote activation of innate immunity	Disruption of trophoblast-endothelial interactions, participation in placental inflammation	(18, 77)

sFlt-1, soluble fms-like tyrosine kinase 1.

**Figure 2:**
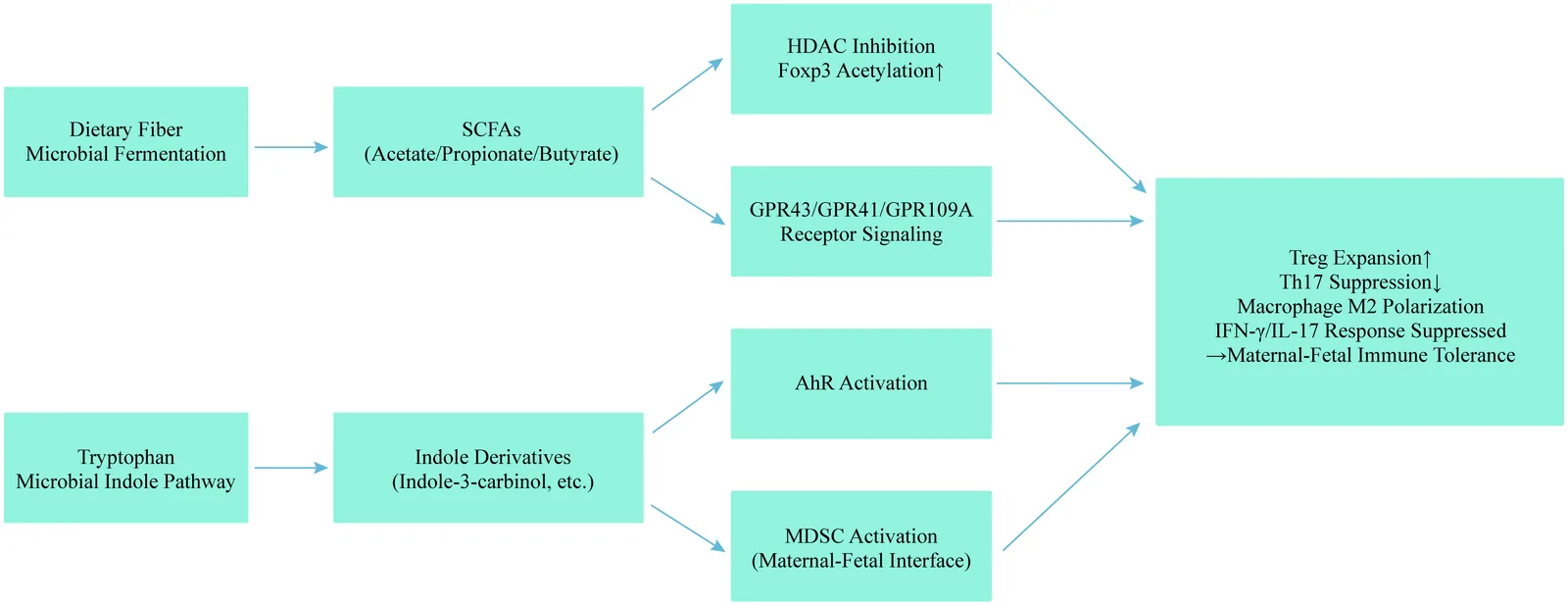
Mechanisms of microbial metabolites in maternal-fetal immune regulation. Dietary fiber fermentation yields SCFAs that signal via GPR41, GPR43, and GPR109A and enhance Foxp3 acetylation. Tryptophan-derived indole metabolites activate AhR at the maternal-fetal interface and MDSCs. Together, these pathways promote Treg expansion, suppress Th17 and IFN-γ responses, and induce M2 macrophage polarization to maintain immune tolerance. ↓ indicates decrease, ↑ indicates increase.

### 5.2 Tryptophan metabolites and the AhR pathway

Tryptophan is metabolized bidirectionally through the host kynurenine pathway and the microbial indole pathway, both of which are deeply involved in pregnancy immune regulation. On the host side, IDO-mediated tryptophan catabolism is a foundational mechanism for maternal-fetal tolerance. IDO expressed by placental syncytiotrophoblasts and decidual immune cells suppresses T cell proliferation by consuming local tryptophan and producing kynurenine (26). Kynurenine and its downstream products are also endogenous ligands for AhR, so the host and microbial tryptophan metabolic pathways converge at the AhR node (27). Dynamic changes in the kynurenine pathway during pregnancy have been documented in multiple clinical studies, including elevated serum kynurenine levels in pregnant women with FGR and oligohydramnios (human association) (78). Human term placentas exposed to LPS or poly I:C show pathological shifts in tryptophan metabolism, with increased kynurenine/tryptophan ratios, suggesting that inflammatory stimuli can hijack this pathway (human ex vivo tissue) (79). The role of the placental tryptophan pathway throughout pregnancy and its relationship with pregnancy outcomes have been comprehensively reviewed (79).

On the microbial side, various intestinal bacteria convert tryptophan into indole, indole-3-propionic acid, indole-3-carbinol, and other derivatives, which regulate host immunity as AhR ligands (27). The maternal-fetal interface study published in Cell confirmed that microbiota-dependent tryptophan derivatives suppress IFN-γ^+^ and IL-17^+^ T cell responses at the maternal-fetal interface through MDSC and RORγt^+^ Treg pathways respectively. Indole-3-carbinol intervention can reshape T cell balance at the maternal-fetal interface in GF mice and reduce fetal resorption; colonization with tryptophan-metabolizing *Lactobacillus murinus* can also partially rescue the phenotype in GF mice, achieving two-level verification from metabolite to single strain (10). Three caveats qualify the weight placed on this study: its causal chain was established in mouse models, with human recurrent miscarriage cases providing associative rather than interventional validation; the relative contributions of gut-educated versus locally induced RORγt^+^ Tregs at the maternal-fetal interface remain to be quantified; and, although the study linked intestinal RORγt^+^ Treg expansion to metabolite-dependent tolerance, it did not directly trace the migration of gut-educated RORγt^+^ Tregs to the maternal-fetal interface, leaving local induction and expansion within the uterine mucosa as plausible and unresolved alternatives (10). The action window of tryptophan metabolites is not limited to pregnancy: maternal indole supplementation during pregnancy can reprogram offspring liver metabolic health through the AhR pathway (mouse) (80); maternal AhR activation can protect newborn rats from necrotizing enterocolitis (81). The microbiota-indole-3-propionic acid-brain axis has also been shown to participate in offspring neurodevelopmental abnormalities caused by maternal microbiota dysbiosis (mouse) (82). The role of tryptophan metabolism in embryo implantation and decidualization has also been specifically reviewed (83). From the developmental origins of health and disease perspective, the programming effects of tryptophan metabolism during pregnancy can extend to offspring adulthood, representing a candidate mechanism linking perinatal microbiota disturbance to long-term disease susceptibility (84) (**Table 1**; **Figure 2**). In sum, tryptophan metabolites are the best-documented molecular link between gut microbiota and maternal-fetal tolerance, although decisive human causal evidence remains to be generated.

### 5.3 Bile acids

Primary bile acids are converted to secondary bile acids in the intestine through bile salt hydrolase (Bacteroides, Bifidobacterium) and 7α-dehydroxylation (specialized Clostridium species). Bile acid metabolites are important mediators through which intestinal microbiota regulate T cell differentiation: Hang et al. found that the bile acid metabolite 3-oxolithocholic acid inhibits T helper 17 cell (Th17) differentiation, while isolithocholic acid promotes Treg differentiation, thereby regulating Th17/Treg balance (70); Song et al. reported that microbiota bile acid metabolites regulate intestinal RORγt^+^ Treg homeostasis through nuclear receptors (71); Campbell et al. proved that bile acid metabolism by specific commensal bacteria promotes peripheral Treg generation (85). Bile acid receptors farnesoid X receptor and TGR5 are widely expressed in myeloid cells, and alterations in microbiota bile acid profiles can regulate macrophage and dendritic cell inflammatory output through these receptors (85). 3-oxolithocholic acid and isolithocholic acid respectively achieve Th17 inhibition and Treg promotion through inhibiting RORγt and enhancing Foxp3 activity. This bidirectional regulation corresponds precisely to the balance most needing maintenance at the maternal-fetal interface (70). Given that RORγt^+^ Tregs are precisely one of the key executors of maternal-fetal interface tolerance, the significance of the bile acid-microbiota-Treg axis in pregnancy warrants in-depth exploration (10). Currently, direct studies linking pregnancy bile acid profiles with maternal-fetal immune phenotypes are lacking, representing a clear gap in this subfield. Future studies should profile the serum and decidual bile acid landscape across gestation in parallel with Treg/Th17 phenotyping, test whether defined secondary bile acids can prevent fetal resorption in dysbiosis models, and determine whether bile acid–nuclear receptor signaling in decidual myeloid cells mediates the metabolite–immune coupling. Because bile acid derivatives are already used clinically in pregnancy (e.g., ursodeoxycholic acid for intrahepatic cholestasis), the bile acid axis may offer one of the most readily translatable entry points among the metabolite lines, provided that dose-window and fetal-safety questions are addressed first. In sum, bile acids are the least studied but potentially most tractable metabolite line, and dedicated pregnancy studies are a clear priority.

### 5.4 Other microbial metabolites

TMAO is produced when intestinal microbiota metabolizes choline and L-carnitine to generate trimethylamine, which is then oxidized by hepatic flavin monooxygenase. Serum TMAO and its source genera are significantly elevated in PE patients and positively correlate with disease severity (human association) (73). Fecal microbiota transplantation experiments confirmed that TMAO can induce PE-like phenotypes, suggesting a causal role (mouse FMT) (73). The biological effects of TMAO are context-dependent: one study found that TMAO derived from endometrial stromal cells, instead, maintains decidualization and prevents recurrent miscarriage, suggesting that the same metabolite can exert opposite effects in different tissue microenvironments (human samples and mouse model) (74). This contrast cautions that equating circulating metabolite levels simply with toxicity or protection may be misleading; tissue source, exposure concentration, and action phase must be incorporated into the interpretation framework. Additionally, microbiota-derived imidazole propionate, p-cresol sulfate, and other uremic toxin-like molecules, as well as branched-chain amino acids, are increasingly receiving attention for their significance in pregnancy complications (86). In sum, TMAO illustrates that the same metabolite can be protective or deleterious depending on tissue source, concentration, and gestational timing.

### 5.5 MAMPs and metabolic endotoxemia

When intestinal barrier function is impaired or microbiota dysbiosis occurs, LPS and other MAMPs from Gram-negative bacteria enter the portal vein and systemic circulation, forming low-grade metabolic endotoxemia. Circulating LPS is presented to the TLR4/MD-2 complex via LPS-binding protein and CD14, activating myeloid differentiation primary response 88-dependent pathways, which then induce release of pro-inflammatory factors such as TNF-α, IL-1β, and IL-6 through NF-κB (17). Trophoblast cells and decidual immune cells both highly express TLR4, making the placenta a highly sensitive target organ for circulating LPS (43). Abnormal activation of trophoblast TLR signaling is associated with placental inflammation, trophoblast apoptosis, and spiral artery remodeling disorders (43). Experimental studies have shown that LPS can induce mitochondrial dysfunction in mouse and human placenta, manifested as decreased oxidative phosphorylation efficiency and increased reactive oxygen species generation, suggesting that energy metabolism collapse is the central executant of placental inflammatory damage (72); anti-β2-glycoprotein I antibodies induce trophoblast pyroptosis and placental inflammation through the TLR4/NOD-like receptor family pyrin domain containing 3 pathway (*in vitro*), providing a molecular explanation for placental lesions in antiphospholipid syndrome (87); metformin alleviates placental inflammation by inhibiting the TLR4/NF-κB pathway to regulate macrophage polarization (rat PE model), suggesting potential for drug repurposing in placental inflammation regulation (88). Chorioamnionitis and intrauterine infection are important causes of PTB, and the decidual inflammatory cascade triggered by MAMPs through ascending infection or hematogenous routes occupies a central position (89). The relationship between intrauterine infection and PTB remains an important topic in perinatal medicine (90). Metabolic status can amplify the placental toxicity of MAMPs: hyperglycemic environments exacerbate placental inflammation induced by Porphyromonas gingivalis LPS (human ex vivo placental explants), providing experimental evidence for the “periodontitis-glucose metabolism abnormality-placental inflammation” triad mechanism (91). Environmental endocrine disruptors also damage the placenta through microbiota-MAMP pathways: bisphenol A exposure induces maternal intestinal and placental apoptosis by disrupting intestinal microbiota homeostasis (ewe model) (92). These studies collectively suggest that the intestinal barrier-microbiota-MAMP axis is a common terminal pathway through which environmental exposures affect placental immunity (**Table 1**). In sum, MAMPs convert intestinal ecological disturbance into placental inflammatory damage, and barrier integrity determines whether microbial signals remain physiologic.

### 5.6 Bacterial extracellular vesicles

Intestinal bacteria release extracellular vesicles carrying LPS, peptidoglycan, proteins, and nucleic acids, which can cross the intestinal mucosa and enter circulation. These vesicles serve as “concentrated carriers” for systemic dissemination of MAMPs (77). Compared to free LPS, vesicle-encapsulated LPS has a longer half-life, stronger tissue penetration, and vesicle surface bacterial proteins determine cellular targeting, explaining how trace microbiota components can produce systemic effects. Extracellular vesicles from periodontal pathogen Porphyromonas gingivalis can disrupt interactions between trophoblast cells and vascular endothelial cells (*in vitro*), providing a mechanistic explanation for the epidemiological association between periodontitis and adverse pregnancy outcomes (18). Oral bacteria such as Fusobacterium nucleatum are associated with placental inflammation and PTB through bacterial extracellular vesicles or hematogenous transmission, and the oral-intestinal-placental axis is gradually receiving attention (77).

### 5.7 Bidirectional communication via placental exosomes

The placenta is the main source of circulating extracellular vesicles during pregnancy. Extracellular vesicles are classified by biogenesis pathway into exosomes (intraluminal vesicles of multivesicular bodies released by exocytosis, 30–150 nm in diameter), microvesicles (direct budding from plasma membrane), and apoptotic bodies. The syncytialization and renewal of syncytiotrophoblasts lead to continuous release of the above vesicles into maternal circulation, with concentrations increasing as pregnancy progresses and further increasing under pathological conditions such as PE (42, 93). Placental exosomes carry placenta-specific proteins (such as placental alkaline phosphatase), miRNAs, and anti-angiogenic factors, which can remotely regulate maternal immune cell function (19). Changes in exosome cargo profiles in pregnancy complications encompass angiogenesis, oxidative stress, and immune regulatory molecules, giving them dual attributes as pathogenic mediators and biomarkers (94). Mechanistic studies have shown that placental exosomal miR-30d-5p promotes dMφ polarization toward an M2-like phenotype by targeting HDAC9 (human samples; *in vitro*) (75); PE-derived trophoblast extracellular vesicles participate in disease development by inducing macrophage polarization toward pro-inflammatory phenotypes (mouse model; *in vitro*) (76). Maternal monocytes show higher capture efficiency for PE-derived vesicles than for normal pregnancy-derived vesicles (human ex vivo), suggesting that pathological vesicles possess stronger immune bias (95). Placental exosomes as messengers connecting maternal and fetal systems have been comprehensively reviewed (19). In PE pathology, the role of extracellular vesicles is particularly prominent, and their mechanisms in disease development have been reviewed (96). Exosomes have unique advantages in “packaging” and “concentrating” low-abundance microbial signals, stably delivering trace microbiota molecules to distal target cells. This characteristic makes them potential amplifiers of dialogue between intestinal signals and placental immunity (4).

### 5.8 Bidirectional transfer of maternal antibodies and cells

Beyond soluble molecules, the maternal immune system establishes connections with the fetus through antibodies and cells. Maternal immunoglobulin G (IgG) is actively transported to fetal circulation through the neonatal Fc receptor, providing passive immunity for newborns, while maternal microbiota-specific IgG can shape offspring mucosal immunity (97). This study found that maternal IgG and IgA against commensal bacteria can suppress excessive early-life T cell responses against microbiota (mouse), thereby “inscribing” maternal microbiota information into offspring immune programs in antibody form. Bidirectional cell migration forms microchimerism, whereby maternal and fetal cells persist long-term in each other’s bodies. Pregnancy can displace pre-existing microchimeric cells and affect reproductive outcomes (mouse) (98). A Science study further showed that microchimeric cells pre-existing in the uterus are competitively replaced by cells from the new fetus during re-pregnancy, suggesting that each pregnancy reshapes the maternal immune memory repertoire. Mobile genetic elements of maternal microbiota can also influence offspring gut microbiota assembly and metabolism through vertical transmission (human cohort), with strain-level mother-infant sharing dominating early-life microbiota establishment (99, 100).

**Table 1** summarizes the systemic immune effects and placental immune outcomes of major intestinal signal molecules. **Figure 2** shows the main molecular mechanisms by which SCFAs and tryptophan metabolites regulate immune cells at the maternal-fetal interface.

## 6 Systemic immune remodeling: gut-mediated immune education

### 6.1 Foundational shaping of the maternal immune system by gut microbiota

The education of host immunity by gut microbiota begins in early life and continues throughout adulthood. Segmented filamentous bacteria adhering to ileal epithelium can induce small intestinal Th17 cell differentiation (mouse), proving that a single commensal bacterium is sufficient to directionally shape adaptive immunity (6); Clostridium clusters IV and XIVa induce colonic Treg generation to maintain mucosal tolerance (mouse) (7). These classical studies established the correspondence between “specific microbiota-specific immune cell lineages,” providing a methodological paradigm for tracing the immunological endpoints of microbiota signals in pregnancy research. During pregnancy, the maternal immune system undergoes remodeling characterized by Treg expansion, Th2 skewing, and adjustment of innate immune activation thresholds, which is continuously calibrated by gut microbiota through metabolites and MAMPs, as systematically reviewed (9). The significance of maternal gut microbiota for offspring immune system development has been extensively studied, and the association between microbiota disturbance and offspring allergy, autoimmunity, and neurodevelopmental abnormalities constitutes an active research direction under the developmental origins of health and disease framework (9, 84). The shaping of maternal immunity by microbiota even extends to offspring: maternal microbiota during pregnancy drives early postnatal innate immune system maturation in offspring through antibody and metabolite pathways, and transient colonization during pregnancy alone can increase offspring intestinal lamina propria mononuclear cell and NK cell numbers and upregulate antimicrobial peptide expression (germ-free mouse) (61); early-life immune development follows microbiota-dependent temporal programs, and its disturbance is associated with long-term immune disease susceptibility (101). The maternal immune system during pregnancy is not passively receiving microbiota signals, but rather shaping microbiota composition through IgA selection, antimicrobial peptide secretion, and intestinal motility changes, forming a bidirectional host-microbiota feedback loop (8, 97). Microbiota metabolites regulate immune cell fate through receptor and epigenetic pathways, representing an important topic in host-microbiota interaction research (16). Microbiota metabolites are also drivers of gut-brain axis communication, and evidence for maternal microbiota disturbance affecting offspring neurodevelopment through pathways such as the microbiota-indole-3-propionic acid-brain axis is continuously accumulating (82).

### 6.2 MDSCs

MDSCs are a heterogeneous population of myeloid cells with immunosuppressive functions, expanding in tumors, infections, and inflammation. Their characteristics include suppressing T cell activation and promoting Treg generation (102, 103). MDSCs can be divided into two major subsets: polymorphonuclear MDSCs and monocytic MDSCs. Their immunosuppressive mechanisms include arginase 1-mediated L-arginine depletion, inducible nitric oxide synthase and reactive oxygen species generation, and inhibitory cytokine release. Pregnancy is one of the few states accompanied by physiological MDSC expansion. Functionally active MDSCs exist in umbilical cord blood (human), participating in the establishment of neonatal immune homeostasis (104); maternal peripheral and decidual MDSCs increase during pregnancy, and their quantitative and functional abnormalities are associated with GDM and RPL (human association) (105, 106). As biomarkers, MDSC frequency and suppressive activity in RPL and recurrent implantation failure have received preliminary clinical evidence support (106). The study published in Cell provided direct evidence for the microbiota-MDSC connection: microbiota-dependent tryptophan derivatives activate MDSCs at the maternal-fetal interface, with MDSCs specifically suppressing IFN-γ^+^ T cell responses; MDSCs were also significantly dysregulated at the maternal-fetal interface in human recurrent miscarriage cases (10). Maternal circadian rhythm disturbance can affect MDSC function through metabolic reprogramming and exacerbate neonatal inflammation (mouse), suggesting that maternal physiological states influence offspring immunity through the MDSC axis (107). Synthesizing existing evidence, the role of MDSCs in pregnancy has distinct dual characteristics: moderate expansion is a necessary link for tolerance establishment, whereas excessive expansion or functional hyperactivation may weaken maternal anti-infection defense and promote an immune escape-like state in pathological pregnancy. Their “dose window” remains to be defined (102, 103). In sum, MDSCs are a physiologic component of pregnancy immunity whose calibration by microbial metabolites is only beginning to be defined.

### 6.3 Regulatory T cell subsets and RORγt^+^ pTreg

Tregs are the central executors of maternal-fetal tolerance. During pregnancy, maternal peripheral and decidual Treg numbers and functions are enhanced. Thymus-derived Tregs and peripherally induced pTregs synergistically maintain tolerance: thymus-derived Tregs mainly recognize self-antigens, while pTregs target fetal alloantigens and microbiota antigens, with complementary division of labor (108). Pregnancy-induced memory Tregs provide rapid tolerance responses upon re-pregnancy (mouse), explaining the clinical observation that parous women have decreased complication rates for pregnancies with the same paternal antigen (24). Progesterone can induce FOXP3^+^ Treg generation, resolving maternal-fetal immune conflict (mouse) (109). Treg functions extend beyond immunosuppression: Treg deficiency leads to decidual spiral artery remodeling disorders, decreased uNK cells, and late pregnancy fetal loss and growth restriction, which can be corrected by adoptive transfer of wild-type Tregs, suggesting that Tregs participate in placental development by regulating vasculature and uNK cells (mouse) (110). Transcriptomic analysis further showed that Treg deficiency leads to significant changes in decidual tissue angiogenesis and inflammation-related gene expression profiles, elevating the “vascular regulatory function” of Tregs from correlation to mechanism (110).

RORγt^+^ Tregs are a pTreg subset induced by gut microbiota, expressing the Th17 lineage transcription factor RORγt while exercising suppressive function. They mainly reside in intestinal lamina propria and are crucial for tolerance to microbiota and food antigens; their homeostasis is regulated by microbiota bile acid metabolites through nuclear receptor pathways (71). The study published in Cell showed that microbiota-dependent tryptophan derivatives promote expansion of intestinal RORγt^+^ Tregs and the accumulation of this subset at the maternal-fetal interface, specifically suppressing IL-17^+^ T cells in mice; this subset was also dysregulated at the maternal-fetal interface in human recurrent miscarriage cases (mouse causal; human association) (10). This discovery directly links gut microbiota-induced specific Treg subsets with placental immune tolerance, representing an important conceptual advance, while leaving the cellular route unresolved: the migration of gut-educated RORγt^+^ Tregs to the maternal-fetal interface was not directly traced, and local induction and expansion within the uterine mucosa remain plausible alternatives (10). The latest advances in Treg functional subsets and key molecules at the maternal-fetal interface have been comprehensively reviewed (111). In sum, RORγt^+^ pTregs are the clearest candidate cellular route by which gut microbiota education may be relayed to the maternal-fetal interface, pending direct lineage-tracing evidence.

### 6.4 Th17/Treg balance

Th17 and Tregs share transforming growth factor-β (TGF-β)-dependent differentiation pathways, and their balance is regulated by IL-6, microbiota metabolites, and inflammatory microenvironments. Normal pregnancy is characterized by Treg dominance, and elevated Th17/Treg ratios are associated with multiple complications including RPL and PE (112). Unexplained RPL patients show elevated peripheral Th17-related cytokines and decreased Treg-related factors (human association) (113). Maternal plasma Th17/Treg cytokines combined with small extracellular vesicles can serve as potential biomarkers for PE (human case-control) (111). Microbiota metabolite intervention has therapeutic potential: SCFAs alleviate embryo loss in miscarriage models by regulating T cell heterogeneity (mouse) (114); bile acid metabolites determine Th17 and Treg differentiation directions from upstream (70). AhR, as a common receptor for microbiota indole ligands, is expressed on both Th17 and Tregs, and ligand type and dose determine T cell differentiation direction, making it the molecular hub for microbiota metabolite regulation of this balance (27, 70). In sum, the Th17/Treg balance is the convergent readout of microbial metabolite input, hormonal milieu, and inflammatory tone.

### 6.5 Macrophage polarization

The plasticity of macrophages makes them important responders to microbiota signals. In the pregnant uterus, macrophage polarization status is finely regulated by local metabolic and hormonal signals. Trophoblast-derived lactate orchestrates dMφ differentiation toward immunoregulatory phenotypes through the tyrosine-protein kinase Src/lactate dehydrogenase A signaling axis (*in vitro* human cells), revealing a reverse signaling pathway of “placental metabolites shaping maternal immune cells” (115); the placental local environment shapes the unique phenotype of dMφ (33), and decidual macrophage arginine metabolism provides an additional layer of functional control (116). Non-microbial pathological stimuli, such as decorin-mediated mitochondrial dysfunction, can also tilt dMφs toward M1-like polarization (117). LPS entering circulation during intestinal microbiota dysbiosis is a powerful driver of M1 polarization, amplifying placental inflammation through the TLR4/NF-κB pathway (rat model) (88). From the perspective of mechanistic hierarchy, microbiota metabolites (SCFAs, bile acids) tend to maintain M2-like phenotypes, while microbiota-derived MAMPs tend to drive M1-like phenotypes. Macrophage polarization status can therefore be regarded as an “immune readout” of intestinal ecological balance (16, 17). HBCs, as placental macrophages, show dynamic transcriptional and epigenetic programs during pregnancy (35), and their interactions with pathogens (such as Listeria monocytogenes) change with gestational stage (human *in vitro*) (118). Phenotypic and functional identification of first-trimester human placental HBCs shows that this cell population highly expresses anti-inflammatory and tissue repair-related genes, serving as gatekeepers of placental local immune homeostasis (34). The discovery that HBCs share transcriptional programs with fetal brain microglia suggests that the developmental programming of placental macrophages may represent general rules for fetal tissue-resident macrophages (mouse), and how they are calibrated by maternal microbiota signals warrants cross-organ comparative research (36); macrophage biology in pregnancy has been comprehensively reviewed (119, 120). In sum, macrophage polarization state can be read as an integrated gauge of intestinal ecological balance at the maternal-fetal interface.

### 6.6 NK cells

The functions of uNK cells extend far beyond traditional cytotoxicity. Beyond promoting trophoblast differentiation and spiral artery remodeling (30), uNK cells maintain decidual homeostasis by inhibiting decidual stromal cell senescence through the TNFSF14-TNFRSF14 axis; impaired mechanisms are associated with spontaneous abortion (human samples and mouse model) (121). The pro-angiogenic and nutrient-sensing functions of early pregnancy uNK cells are indispensable for placentation, with secreted vascular endothelial growth factor, placental growth factor, and chemokines directly participating in spiral artery lumen expansion (28). Abnormal uNK cell numbers and function are important immunological features of recurrent miscarriage and recurrent implantation failure, and their clinical assessment value has accumulated multi-center evidence (31). Abnormal activation of maternal NK cells can also affect offspring across generations: maternal NK cells drive offspring neuroimmune abnormalities through aberrant secretion programs, suggesting that maternal immune cell functional status is an independent variable for offspring neurodevelopment (mouse) (122). Direct evidence for gut microbiota regulation of NK cells exists: maternal gut microbiota dysbiosis damages placental NK cell IFN-γ secretion by disrupting placental carbohydrate metabolism, and glucose supplementation can restore their function and reduce fetal resorption, suggesting the reversibility of the microbiota-metabolism-NK axis (mouse) (123). The implication of this study is that placental immune cell effector functions depend on local metabolic supply, and microbiota dysbiosis can induce functional paralysis of placental NK cells through “metabolic deprivation” pathways, providing a theoretical anchor for metabolite supplementation interventions. Treg regulation of uNK cells further couples adaptive and innate immunity in the decidua (mouse) (110). The role of NK cells in pregnancy and its complications has been specifically reviewed (119). In sum, uNK function couples immune tolerance to local metabolic supply, a coupling that microbial dysbiosis can disrupt and metabolite supplementation can partially rescue.

### 6.7 Other immune cells and newly discovered regulatory subsets

Regulatory KIR^+^CD8^+^ T cells increase in peripheral blood during mid-pregnancy and can selectively suppress maternal CD8^+^ T cell alloresponses against fetal-specific antigens (human), providing new evidence for CD8 lineage participation in maternal-fetal tolerance (38). Human type 2 conventional dendritic cells acquire tolerogenic phenotypes through homeostatic maturation programs, participating in the remodeling of antigen presentation patterns during pregnancy, suggesting that the antigen presentation link itself is a regulatory node for tolerance establishment (120). Regarding B cells, maternal microbiota-induced IgG is transferred to offspring through neonatal Fc receptor and regulates their mucosal immunity (mouse), so maternal antibodies become carriers for cross-generational transmission of microbiota information (97). These findings suggest that the education of systemic immunity by intestinal signals is multi-lineage and multi-layered, and the placental immune microenvironment is a local projection of this systemic education. Viewing the various lineages together, microbiota metabolites simultaneously act on myeloid and lymphoid cells through three molecular pathways: AhR, G protein-coupled receptors, and epigenetics, ultimately converging at the maternal-fetal interface into a tolerance program centered on suppressing IFN-γ and IL-17 (10, 16). In sum, microbial education of systemic immunity is multi-lineage, and the decidual microenvironment is its local projection.

## 7 Remodeling outcomes of the placental immune microenvironment

### 7.1 Establishment and maintenance of immune tolerance

Comprehensively depicted intestinal signals ultimately converge at the maternal-fetal interface as a tolerant microenvironment through systemic immune transmission. Single-cell atlas studies have charted the cellular anatomical structure of the maternal-fetal interface (human), revealing molecular networks of cell interactions between decidua and placenta, providing high-resolution maps for locating cellular targets of microbiota signals (39). The coupling of energy metabolism and maternal-fetal tolerance during decidualization suggests that metabolic reprogramming of immune cells is the deep mechanism for tolerance establishment (mainly mouse evidence) (124, 125). TGF-β signaling runs through pregnancy inflammation, placental health, and PE development, serving as an important molecular node connecting microbiota, immunity, and placenta: TGF-β both drives Treg differentiation and decidualization, and participates in inflammation-fibrosis imbalance in PE placenta (human and mouse evidence; review-level synthesis) (126). Immunometabolic perspectives on T cell tolerance studies show that decidual T cell activation thresholds are gated by local nutrient and metabolite concentrations, providing mechanistic rationale for remote regulation of decidual T cells by microbiota metabolites (125). From an integrated perspective, maintenance of the tolerant microenvironment depends on three levels of redundant design: antigen level (trophoblast non-classical HLA expression), cellular level (synergy of Tregs, MDSCs, M2-like macrophages, and uNK), and molecular level (IDO, TGF-β, SCFAs, tryptophan derivatives). This synthesis rests on mouse causal studies and human association data; microbiota signals can simultaneously act on multiple levels, explaining why microbiota disturbance can produce systemic pregnancy consequences (10, 26, 40).

### 7.2 Microbiota regulation of placental development and angiogenesis

The effect of intestinal microbiota on the placenta extends beyond immunity. Gnotobiotic mouse studies show that maternal microbiota absence leads to disordered vascularization in the placental junctional zone and labyrinth layer, decreased fetal weight (gnotobiotic mouse), and that pre-pregnancy colonization with conventional microbiota can rescue the above phenotypes. Mechanistically, GF dams show compensatory elevation of pro-angiogenic proteins (angiopoietin 2, fibroblast growth factor 2, etc.) in the placental labyrinth layer while endothelial cell density decreases, suggesting impaired angiogenic signal transduction (127). Maternal microbiota promotes placental vascular endothelial function and placental growth through SCFAs (mouse) (65). Functional products of specific commensal bacteria also participate: extracellular vesicles from *Akkermansia muciniphila* can promote placentation and alleviate PE-like phenotypes (mouse), with mechanisms involving enhanced trophoblast invasion capability and improved placental angiogenesis (128). The Treg-uNK-spiral artery axis directly connects immune cells with placental vascular remodeling (110). Spiral artery remodeling is the rate-limiting step for placentation, and analysis of its immune regulatory mechanisms provides an anatomical anchor for understanding microbiota-immunity-vessel tripartite interactions (129).

### 7.3 Placental defense and inflammation: a double-edged sword

Comprehensively summarized immune activation at the maternal-fetal interface must be precisely calibrated: excessive inflammation leads to fetal loss and placental damage, while insufficient defense increases infection risk. Innate immune responses to pathogens at the maternal-fetal interface possess tissue specificity, and a Nature Reviews Immunology review of the division of labor among trophoblasts, decidual immune cells, and HBCs in antiviral and antibacterial defense (130). Listeriosis caused by Listeria monocytogenes transplacental transmission is a typical representative of pregnancy infection, and its vertical transmission efficiency is directly related to the phagocytic and killing capacity of HBCs (human *in vitro*), which evolves with gestational stage (118). The decidual-chorionic inflammatory cascade triggered by intrauterine infection is the main pathological pathway for PTB (human clinical and histopathological evidence) (89, 131). Trophoblast defense against viruses depends on the synergy of type I interferons and autocrine interferon lambda, with continuous low-level interferon signals from syncytiotrophoblasts constituting a pre-activated defense line for the fetus against blood-borne infections (130). Intestinal microbiota indirectly determines placental response intensity to infection by regulating systemic immune activation thresholds (mouse), constituting the upstream regulation of this “double-edged sword” (123). This perspective also explains why antibiotic abuse-induced microbiota disturbance may simultaneously weaken both immune tolerance and anti-infection defense dimensions.

## 8 Axis imbalance under pathological conditions: major pregnancy complications

### 8.1 PE

PE is characterized by new-onset hypertension after 20 weeks of gestation accompanied by proteinuria or multi-organ dysfunction, and is one of the leading causes of maternal mortality worldwide (**Figure 3**). Representative evidence linking gut microbiota, metabolic, and immune alterations to major pregnancy complications is summarized (**Table 2**). Its pathological core is shallow placental implantation and spiral artery remodeling disorders leading to placental ischemia, hypoxia, and release of anti-angiogenic factors (137). Vascular endothelial dysfunction is the direct execution mechanism of PE maternal syndrome, involving decreased nitric oxide bioavailability, elevated endothelin-1, and glycocalyx damage (138). Single-cell transcriptomics studies have revealed two molecular subtypes of PE: one primarily characterized by maternal vascular dysfunction, and the other by intrinsic placental defects, suggesting that PE heterogeneity requires stratified intervention (139). Recent studies indicate that gut microbiota dysbiosis is one of the upstream driving factors of PE.

**Figure 3:**
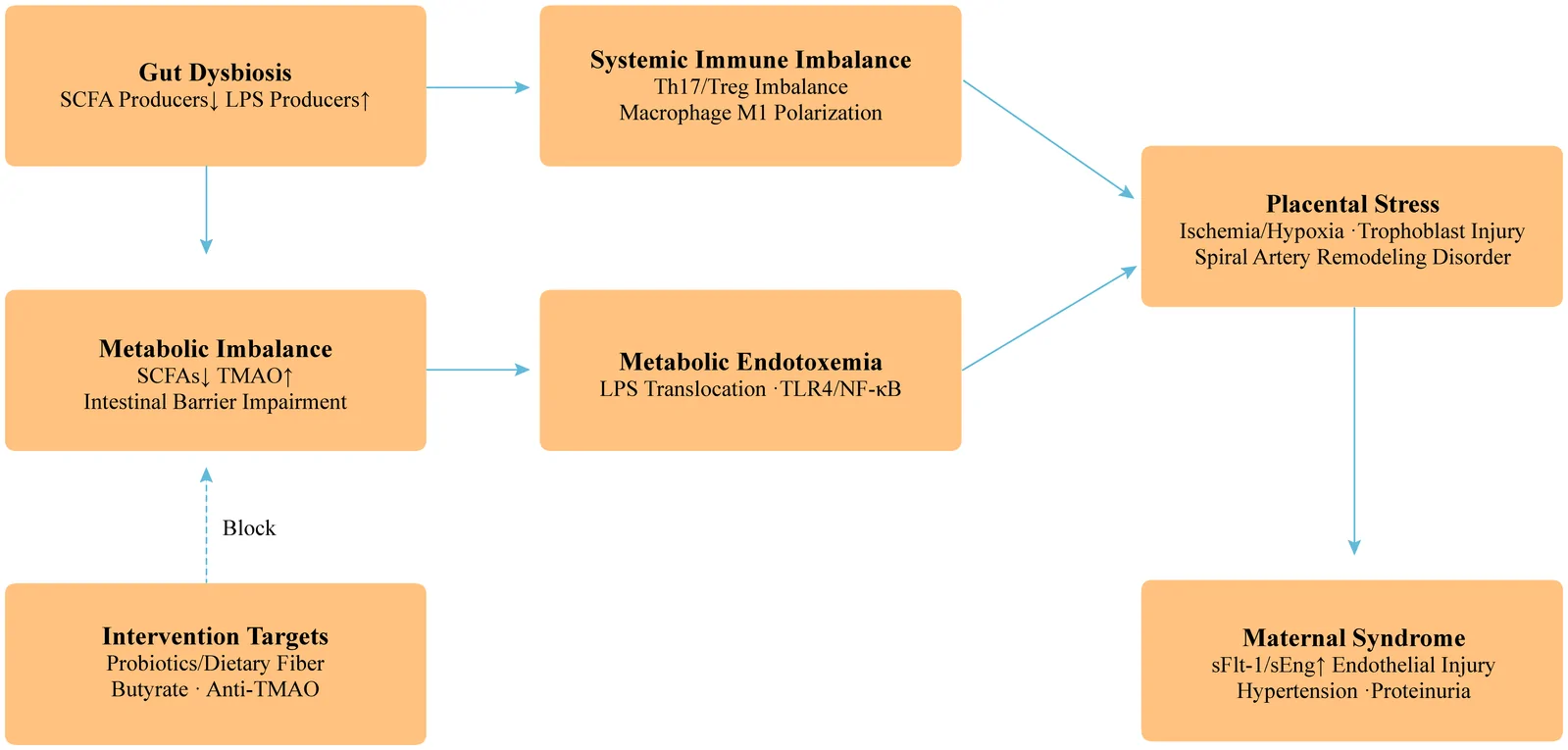
Pathological cascade of preeclampsia originating from gut dysbiosis. Reduced SCFA producers and increased LPS producers trigger systemic immune imbalance and metabolic endotoxemia. These insults cause placental stress characterized by ischemia and trophoblast injury. Consequently, maternal syndrome develops with elevated sFlt-1, endothelial damage, hypertension, and proteinuria. Interventions targeting microbiota and metabolites may block this cascade. ↓ indicates decrease, ↑ indicates increase.

**Table 2:** Microbiota-metabolic-immune alterations in pregnancy complications.

Disease	Microbiota alterations	Metabolic alterations	Immune abnormalities	Placental outcomes	References
PE	Decreased SCFA-producing bacteria such as F. prausnitzii, increased LPS-producing bacteria (human association; causality shown by mouse FMT)	Decreased SCFAs, elevated TMAO	Th17/Treg imbalance, suppressed macrophage immunoregulatory function	Spiral artery remodeling disorders, placental ischemia/hypoxia, elevated sFlt-1	(11, 73, 132)
GDM	Microbiota dysbiosis appearing months before diagnosis	SCFA profile changes, abnormal bile acids and branched-chain amino acids	Microbiota-induced systemic inflammation, MDSC functional abnormalities	Exacerbated placental TNF-α-related inflammation	(12, 48, 133)
RPL	Intestinal and reproductive tract microbiota dysbiosis	Dysregulation of microbiota-dependent tryptophan derivatives	Decreased CCR8^+^ decidual Tregs, MDSC and RORγt^+^ Treg dysregulation	Decidual homeostasis disruption, embryo loss	(10, 13, 37)
PTB	Gut microbiota characteristics can predict PTB risk, loss of vaginal Lactobacillus dominance	Enrichment of 17β-estradiol-degrading C. innocuum	Intrauterine infection and intra-amniotic inflammation	Chorioamnionitis, fetal inflammatory response syndrome	(14, 89, 134)
FGR	Maternal microbiota absence or dysbiosis	Altered microbiota metabolite profiles	dMφ and HBC functional abnormalities	Placental vascularization disorders, decreased fetal weight	(127, 135, 136)

Two-sample Mendelian randomization analysis supports a causal association between gut microbiota and PE (132). Such MR estimates rest on the usual instrumental-variable assumptions (relevance, independence, and exclusion restriction), which are only partially testable for microbiome exposures because microbial taxa are modifiable, polygenic, and environmentally confounded; we therefore interpret the MR result as supportive, not definitive, evidence of causality. In population studies, PE patients show decreased SCFA-producing microbiota (such as *Faecalibacterium prausnitzii*) and increased LPS-producing bacteria, accompanied by decreased SCFA levels and elevated TMAO (human association) (73, 140). A scoping review has summarized the evidence on SCFAs and PE (141); systematic reviews of microbiota dysbiosis and PE risk also support the robustness of these associations (142). PE patients’ microbiota characteristics have potential diagnostic value, and the feasibility of microbiota-derived SCFAs as PE diagnostic biomarkers has been evaluated in clinical samples (human case-control) (140). Integrative reviews on mechanisms, biomarkers, and probiotic interventions for microbiota dysbiosis note that the strength of evidence in PE microbiota research is progressing from association toward intervention validation (143, 144).

In mechanistic studies, work published in Circulation Research provided causal evidence—via fecal microbiota transplantation into mice—that PE patient microbiota dysbiosis drives disease through the “microbiota-macrophage-trophoblast” axis: mice transplanted with PE patient microbiota developed PE-like phenotypes, with microbiota dysbiosis damaging the placenta by suppressing macrophage immunoregulatory function, exacerbating trophoblast inflammation and invasion disorders. Supplementation with F. prausnitzii or its metabolite butyrate could alleviate the above phenotypes (11). This study represents a milestone for causal evidence of microbiota in the PE field, and its experimental logic of “patient microbiota transplantation-phenotype transfer-single bacterium supplementation” has established a standard for similar studies. TMAO is elevated through the microbiota-host co-metabolism pathway and drives PE progression, and targeting TMAO can alleviate animal model phenotypes (73). Sodium butyrate supplementation improves intestinal barrier and microbiota composition, alleviating rat PE-like phenotypes (69); endogenous AhR ligands can induce PE-like phenotypes and disrupt endothelial function, suggesting that imbalance in microbiota indole metabolism also participates in PE development (145, 146). At the immune level, PE exhibits systemic Th17/Treg imbalance, regulatory cell functional defects, and a vicious cycle of endothelial dysfunction (147, 148); TLR7 overexpression participates in PE development by suppressing trophoblast function (mouse model) (149); the chromosome 19 miRNA cluster protects trophoblasts from excessive innate immune activation (*in vitro*) (150). Maternal microbiota dysbiosis also damages placental NK cell function through placental glucose metabolism disorders, and glucose supplementation can reverse this phenotype, directly connecting PE microbiota mechanisms with metabolic intervention (123). It should be emphasized that the causal cascade from dysbiosis to PE-like phenotype is currently demonstrated in animal models; in humans, the corresponding evidence is associative (microbiota signatures, metabolite levels, MR), and the cascade should be read as a plausible, partially validated framework rather than an established human causal chain.

The ischemic placenta releases sFlt-1 and soluble endoglin (sEng), which antagonize vascular endothelial growth factor and placental growth factor, leading to maternal vascular endothelial dysfunction. Placental stress further amplifies maternal-fetal interface damage. The sFlt-1/placental growth factor ratio has become a clinical biomarker for PE prediction and stratification (151, 152). Circulating extracellular vesicles and neutrophil extracellular traps synergistically exacerbate endothelial damage (human association) (153); PE-derived extracellular vesicles participate in systemic inflammation through complement activation and monocyte capture (95, 154). The discovery of the exosome-complement axis incorporates humoral immunity into the amplification circuit of PE placental signals, with complement activation products both directly damaging endothelium and further activating monocytes and neutrophils through complement receptors (human ex vivo) (154). Long non-coding RNA-level regulation has also been analyzed: TLR8 antisense RNA induces PE-like phenotypes through the TLR8/signal transducer and activator of transcription 1 axis (*in vitro* and mouse PE model) (155). Connecting microbiota mechanisms with the classical PE pathological framework, one can outline a four-level cascade of “microbiota dysbiosis-systemic inflammation-placental stress-maternal syndrome”: microbiota dysbiosis provides upstream drive, systemic inflammation serves as an amplifier, placental ischemia, hypoxia, and anti-angiogenic factor release constitute the hub, and maternal vascular endothelial dysfunction is the terminal phenotype (11, 137). Amniotic fluid-derived exosomal miR-146a-5p can alleviate PE phenotypes by inhibiting the HIF-1α/FLT-1 axis (animal model), suggesting the bidirectional effects of exosome cargo (156). The potential value of F. prausnitzii, a representative SCFA-producing bacterium, and of its extracellular vesicle-mediated mechanisms in PE prevention and treatment has also received attention (mouse) (157). The regulatory network and clinical prospects of the gut-placental axis in PE have been reviewed (158). At the clinical translation level, the screening system combining sFlt-1/placental growth factor ratio with uterine artery Doppler has been used for PE risk stratification (152), and microbiota biomarkers are expected to serve as a supplementary dimension to this system: microbiota characteristics do not depend on imaging equipment, can be dynamically monitored, and directly reflect upstream mechanisms. Their combination model with angiogenic biomarkers is worth validating in prospective cohorts (140, 142). In sum, the four-level cascade from dysbiosis to maternal syndrome is mechanistically coherent and supported by animal causality plus human association; its human causal links remain under validation.

### 8.2 GDM

GDM is the most common metabolic complication during pregnancy, and its relationship with gut microbiota has received support from multi-omics and causal evidence. A prospective cohort study published in Gut profiled the gut microbiota, metabolome, inflammatory factors, and clinical data of 394 pregnant women in early pregnancy (before GDM diagnosis), and found that GDM patients had microbiota dysbiosis and microbiota-induced inflammation months before diagnosis, and that microbiota-based models could predict GDM occurrence in advance; fecal microbiota transplantation experiments in germ-free mice further confirmed the causal role of the microbiota in the disease (12), with the human cohort establishing association and prediction. The clinical significance of this study lies in advancing the GDM warning window from the oral glucose tolerance test at 24–28 weeks of gestation to early pregnancy, gaining time for primary prevention. Maternal GDM is accompanied by altered gut microbiota composition and is associated with offspring health outcomes; maternal GDM microbiota influences offspring microbiota establishment and metabolic phenotypes through vertical transmission (human cohort) (159). Integrative metagenomic and metabolomic analyses reveal that microbiota-derived “multiple hits” participate in GDM development (human case-control), including SCFA profile changes, bile acid metabolism abnormalities, and elevated branched-chain amino acids (133). Gut microbiota and serum metabolic alterations in recurrent GDM patients suggest that microbiota memory may participate in cross-pregnancy recurrence of the disease (human association) (160). Systematic reviews and meta-analyses have confirmed the cross-cohort consistency of GDM-associated microbiota alterations, and noted that microbiota alterations can extend to offspring gut microbiota establishment (161, 162).

Mechanistically, microbiota dysbiosis couples metabolism with placental inflammation through two pathways: first, decreased SCFAs weaken the intestinal barrier, promoting LPS translocation and metabolic endotoxemia, exacerbating insulin resistance; second, the placenta is sensitive to systemic inflammation, with TNF-α and other factors amplifying inflammation locally and damaging placental function (48). Characteristic changes in circulating SCFA profiles in GDM patients (68) and molecular details of host-microbiota interactions (163, 164) have been reported. Hyperglycemic environments themselves can also exacerbate pathogen-associated molecular pattern stimulation of the placenta: Porphyromonas gingivalis LPS induces stronger placental inflammation under hyperglycemic conditions (human ex vivo), suggesting a synergistic amplification effect between GDM and subclinical infection (91). The molecular mechanisms and interactions of the placenta-gut microbiota axis in GDM have been specifically reviewed (165). MDSC functional abnormalities also participate in GDM immune imbalance (human association) (105). Regarding interventions, early pregnancy initiation of probiotics and dietary fiber in fermented milk supplementation showed potential for improving metabolic indicators in randomized controlled trials (166). The gut microbiota–SCFA–placental inflammation mechanism has been demonstrated in rodent GDM models; corresponding human data remain associative. In sum, GDM offers the strongest human evidence base among these complications, with animal models supplying mechanism.

### 8.3 RPL

RPL is defined as two or more consecutive pregnancy losses, with approximately half of cases having unknown etiology, and immune factors are considered to occupy an important position (167, 168). The immune pathology of RPL has multi-layered characteristics: local decidua manifestations include NK cell subset imbalance, macrophage polarization abnormalities, and decreased Treg numbers and function; systemic manifestations include elevated Th17/Treg ratios and regulatory cell subset defects. These two levels mutually amplify through the microbiota-metabolism axis (37, 113). Immunological evaluation of RPL covers decidual NK cells, macrophages, Treg/Th17 balance, and autoantibody profiles, and the evidence base for clinical management strategies has significantly strengthened in recent years (169). The intestinal-decidual immune-metabolism interaction is an emerging direction in RPL research: microbiota dysbiosis creates a “decidual hostility” microenvironment through metabolic and immune dual pathways, driving RPL development (13). The study published in Cell directly observed MDSC, RORγt^+^ Treg, and microbiota-dependent tryptophan derivative dysregulation at the maternal-fetal interface in human RPL cases, providing human evidence for the microbiota-immunity-miscarriage chain (10).

At the cellular mechanism level, RPL patients show decreased CCR8^+^ decidual Tregs, and adoptive transfer of this subset can rescue fetal loss in animal models (human; mouse rescue) (37); TNFSF14^+^ decidual NK cells prevent miscarriage by restricting leucine-mediated decidual stromal cell senescence (human and mouse), and high-leucine diets increase embryo loss rates in mice (121); stagnation of endometrial decidualization reaction determines recurrence risk of miscarriage (human), suggesting that decidual quality is a prerequisite for embryo implantation success (170). Macrophage polarization imbalance is closely related to RPL (171, 172). Oxidative stress and autophagy abnormalities also participate in decidual microenvironment deterioration in RPL, and autophagy dialogue between immune cells and decidual cells has become a new mechanistic research direction (173, 174). Regarding environmental factors, the interactive effect of bisphenol F exposure and TLR4 gene polymorphism increases unexplained miscarriage risk (human association), suggesting the existence of environment-gene-immune triad interactions (175). Microbiota interventions show potential: SCFAs alleviate miscarriage model phenotypes by regulating T cell heterogeneity (mouse) (114); butyrate-producing bacteria supplementation can reduce PTB and miscarriage risk by correcting microbiota dysbiosis (176). Endometrial and vaginal microbiota abnormalities also participate in RPL (human association) (177, 178). The association between chronic inflammatory placental disease spectrum (such as chronic villitis, intervillositis) and recurrent adverse pregnancy outcomes suggests that local placental immune pathology can serve as a histological clue for RPL (human histopathology) (179). The relationship between HLA-G expression abnormalities and RPL has been reviewed (41). Regarding clinical treatment, a randomized controlled trial of prednisone improving live birth rates in recurrent implantation failure patients provided high-quality evidence for immune intervention (180); clinical guidelines have established clear boundaries for standardized evaluation and immune treatment indications for RPL (181). Human decidual studies provide the cellular correlates, whereas the microbiota-driven causal pathways have been modeled mainly in animals. In sum, RPL is where human cellular evidence (decidual Tregs, MDSCs) and microbiota mechanisms intersect most directly.

### 8.4 PTB

PTB is the leading cause of neonatal death, with infection and inflammation as its core pathological pathways (89). Intra-amniotic inflammation can be triggered by ascending infection or sterile inflammation (danger signal-driven), and its immunobiological characteristics determine different indications for anti-inflammatory and anti-infection strategies (90). The value of maternal gut microbiota in PTB prediction has made important progress: analysis of two independent cohorts totaling 5313 Chinese pregnant women identified 11 genera and 1 species associated with PTB. Microbial risk scores can effectively distinguish short gestational cycles and high-risk PTB populations (human cohort; mouse validation) (14). Among them, Clostridium innocuum exhibits 17β-estradiol-degrading activity *in vitro* and in pregnant mice; the responsible gene (k141_29441_57), validated by heterologous expression in E. coli, was enriched in women with PTB, providing clues for the microbiota-hormone-PTB mechanism, and microbial risk scores interact with host polygenic susceptibility to amplify risk (14). Regarding vaginal microbiota, systematic reviews and network meta-analyses support the association between vaginal microbiota characteristics and PTB risk, with the shift from Lactobacillus-dominant types to multiple anaerobes coexisting types being a marker of elevated risk (human meta-analysis) (134). The role of extracellular vesicles in spontaneous PTB has been reviewed (182). Mechanistic studies of butyrate-producing bacteria alleviating PTB by correcting microbiota dysbiosis suggest intervention feasibility: supplementation with butyrate-producing bacteria can reduce PTB rates in microbiota-dysbiosis-induced pregnant mice and improve placental inflammation indicators (176). The relationship between chorioamnionitis and neonatal outcomes highlights the cross-generational consequences of placental inflammation, with fetal inflammatory response syndrome increasing risks of bronchopulmonary dysplasia and brain injury (human clinical cohort) (131). In sum, PTB links the axis to ascending and hematogenous infection routes, and microbiota-based risk stratification is nearing clinical application.

### 8.5 FGR

Research on microbiota mechanisms in FGR has grown rapidly in recent years. Maternal heat exposure induces FGR through the gut-placenta axis, and melatonin can alleviate this phenotype by regulating microbiota (mouse), with mechanisms involving restoration of microbiota metabolite profiles and reduction of placental oxidative stress (135); maternal galectin-3 deficiency induces FGR through altering the gut-placenta axis (mouse), suggesting functional coupling between maternal immune regulatory molecules and microbiota (183). Gnotobiotic mouse studies directly prove that microbiota absence leads to placental vascularization disorders and decreased fetal weight (127). The microbiota-indole-3-propionic acid axis-mediated placental and neurodevelopmental abnormalities expand the mechanism spectrum of FGR (mouse) (82). Pathological changes in placental tryptophan metabolism in FGR pregnancies also have clinical evidence (human association) (78). Changes in dMφs and HBCs in FGR suggest immune cell participation in placental insufficiency (human association) (136). Environmental exposures also participate in FGR through microbiota pathways: environmental pollutants such as bisphenol A induce placental apoptosis through microbiota dysbiosis (ewe model) (92). Common characteristics of microbiota alterations under multiple disease states during pregnancy suggest that FGR and other complications may share upstream mechanisms of the microbiota-inflammation axis (86). As with the other complications, FGR causality is currently anchored in animal models, with human evidence largely associative. In sum, FGR mechanisms converge on placental vascular insufficiency, with microbiota-derived metabolites as upstream modulators.

### 8.6 Value of large animal models in axis mechanism research

Existing mechanistic evidence mainly comes from rodent models, but mouse placenta is of the hemochorial type and the gestation period is short, differing from human placenta in diffusion efficiency and immune composition. Pigs offer unique comparative value for human medicine in gestation length, placental structure (epitheliochorial type), gut microbiota composition, and immune system development. Fetal pig studies show that SCFAs can regulate fetal intestinal permeability, motility, and immune-related gene expression, providing a basis for using pig models to study the perinatal effects of microbiota metabolites (67). Livestock reproductive disorder studies also suggest that the interaction between intrauterine inflammation and microbiota imbalance is conserved across species, with cattle and pig endometritis microbiota-immunity research providing comparative references for understanding human pregnancy intrauterine inflammation (184). The establishment of immunodeficient pig models enables *in vivo* study of the effects of immune system reconstruction on pregnancy microbiota-immunity interactions, a direction whose complementarity with mouse GF models is worth noting. Using pigs and other large animals to construct pregnancy microbiota intervention and immune monitoring systems can provide platforms for causal validation and preclinical safety evaluation of the gut-placental axis, compensating for rodent deficiencies. Because most of the mechanistic evidence reviewed above derives from rodents, the question of large-animal model validity is not peripheral but central to translation. These considerations feed directly into the clinical translation section that follows: large-animal platforms are likely to be prerequisites for safety evaluation of microbiota-based interventions before human trials.

## 9 Clinical translation prospects

### 9.1 Probiotics and synbiotics

Probiotic intervention is the most active direction in gut-placental axis translational research (**Table 3**). Randomized double-blind placebo-controlled trials have evaluated the impact of probiotic supplementation during pregnancy on maternal and infant health, showing safety and partial metabolic benefits (185). Systematic reviews and meta-analyses of probiotics/synbiotics improving glucose and lipid metabolism in GDM populations have provided pooled evidence (186). A multi-center randomized placebo-controlled trial evaluated the preventive value of probiotics for Group B Streptococcus colonization during pregnancy (187). Systematic reviews of probiotics, prebiotics, and synbiotics during pregnancy and lactation on intestinal microecology suggest that effects are strain and dose specific (191). However, the available trials remain small. The probiotic trial in healthy pregnant women randomized 180 participants (185), the multicenter Group B Streptococcus prevention trial randomized 267 women (187), and the first-trimester GDM prevention trial in overweight and obese women randomized 356 participants across three arms (166). Moreover, the largest pooled evidence to date, a meta-analysis of 11 RCTs with 779 participants, was built on individual trials enrolling only 20 to 60 women each, most of them from a single country (186). These trials were powered for surrogate outcomes such as infection frequency, GBS colonization, or glycemic indices rather than hard obstetric endpoints such as PE, PTB, or FGR, and the intervention strains were highly heterogeneous. Therefore, high-quality randomized controlled trials using placental immune phenotypes as intermediate endpoints are urgently needed (185, 186).

**Table 3:** Interventions targeting the gut-placental axis: research status.

Intervention strategy	Representative measures	Mechanistic basis	Research stage	References
Probiotics/ synbiotics	Lactobacillus, Bifidobacterium preparations	Correct microbiota dysbiosis, restore SCFA production	RCTs of 180 to 356 participants each; hard endpoint evidence insufficient	(185–187)
Dietary fiber/prebiotics	Highly fermentable fibers (konjac glucomannan, etc.)	Act through microbiota-SCFA-placental inflammation axis	Animal mechanisms clear, population evidence accumulating	(48, 188, 189)
Postbiotics and metabolite supplementation	Butyrate, indole metabolites, butyrate-producing bacteria preparations	Directly supplement effector molecules, bypass microbiota individual differences	Animal model validation, preclinical stage	(69, 80, 176)
Exosome therapy	Engineered extracellular vesicles, bacterial vesicles	Targeted delivery of immune regulatory cargo	Preclinical research	(128, 156, 190)
Microbiota marker prediction	Microbial risk scores, microbiota-SCFA profiles	Early identification of high-risk populations, advancing intervention windows	Large cohort validation stage	(12, 14, 140)

### 9.2 Diet and dietary fiber

Dietary fiber is the most economical means of regulating the microbiota-SCFA axis. High-fiber diets can alleviate maternal obesity-induced offspring cognitive and social dysfunction, with effects dependent on SCFAs produced by microbiota fermentation (mouse) (188). The mechanism by which dietary fiber supplementation reduces GDM susceptibility through the microbiota-SCFA-placental inflammation axis has been clarified in animal models (48). Prenatal dietary fiber supplementation reducing preterm birth risk and improving glycemic control through microbiota mechanisms has received consistent support in population and experimental studies (human RCT; animal models) (192). Fermentation spectra differ significantly among different fiber sources: highly fermentable fibers such as konjac glucomannan preferentially enrich Lachnospiraceae, while resistant starch preferentially enriches Ruminococcaceae, with fiber choice determining SCFA profile composition (48). Maternal prebiotic supplementation can alter gut microbiota and SCFA profiles during pregnancy and lactation (human trial) (189). The impact of pregnancy nutrition on gut microbiota and fetal development has been comprehensively reviewed (193). The advantage of dietary intervention lies in safety and accessibility, while its limitation is its dependence on the individual microbiota baseline: individuals with low fiber fermentation capacity may not obtain equivalent SCFA elevation from the same dietary plan, suggesting that future stratified nutrition strategies based on microbiota baseline should be developed (48, 189).

### 9.3 Postbiotics and metabolite supplementation

Postbiotics refer to inactive bacterial components or metabolite preparations, avoiding safety concerns of live bacterial preparations. Direct supplementation of butyrate and indole metabolites has shown efficacy in correcting placental immune imbalance in animal models (69, 80). Supplementation with butyrate-producing bacteria or their metabolites can reduce PTB and miscarriage risks (mouse) (176). Targeting the TMAO pathway is a new direction for PE prevention and treatment, and strategies such as microbial trimethylamine lyase inhibitors developed in cardiovascular disease research can be directly borrowed for the perinatal field (cardiovascular preclinical evidence; not yet tested in pregnancy) (73). The mechanisms and effects of prebiotics, probiotics, and postbiotics on maternal-fetal health have been systematically compared (194). Another advantage of postbiotics is clear composition and batch-to-batch controllability, better conforming to pharmaceutical regulatory logic; butyrate sustained-release preparations, indole AhR ligands, and microbiota bile acid modulators remain at the preclinical stage (69, 70, 80).

### 9.4 Extracellular vesicle therapy

Placental and stem cell-derived exosomes have targeted delivery and immune regulation advantages. The application prospects of engineered extracellular vesicles in female reproductive and pregnancy-related diseases are broad (190). A study showing that amniotic fluid-derived exosomal miR-146a-5p alleviates PE phenotypes suggests the therapeutic value of exosome cargo (animal model) (156). The discovery that extracellular vesicles from commensal bacterium *Akkermansia muciniphila* promote placentation introduces “bacterial vesicle drugs” into the perinatal intervention horizon (mouse) (128). Key bottlenecks for vesicle therapy lie in placental targeting efficiency and cargo consistency: medication during pregnancy must balance maternal tolerance and fetal safety, and optimization of surface modification and dose windows is a necessary step before clinical translation (190).

### 9.5 Existing challenges

Clinical translation in this field still faces multiple challenges. First, causal evidence is insufficient: most population studies are observational designs, and the temporal relationship between microbiota alterations and disease is difficult to establish. Methodological pitfalls of low-biomass samples must be guarded against (55, 142). Second, individual heterogeneity is substantial: diet, geography, ethnicity, and genetic factors all influence microbiota baseline, making unified intervention schemes difficult to implement. Research suggesting that social structural factors influence pregnancy outcomes through microbiota pathways indicates that microbiota interventions must be evaluated in real-world contexts (195). Third, safety concerns remain: long-term safety data for live bacterial preparations during pregnancy are limited, and the long-term impact of microbiota interventions on offspring immune development remains unclear (185). The choice of intervention window also lacks consensus: early pregnancy intervention can affect placentation, while late pregnancy intervention is closer to metabolic outcomes, and different indications may require different windows (12, 65). Fourth, mechanism depth is insufficient: direct target cells and receptor mechanisms of intestinal signals within the placenta are mostly unclear, hindering precise intervention design (4). Future integration of multi-omics, organoids, and gnotobiotic animal models is needed to drive translation from association to mechanism to clinic (15). Trophoblast organoids and placenta-on-chip *in vitro* systems can already be used to simulate direct effects of microbiota metabolites on trophoblast differentiation (30), while fecal microbiota transplantation combined with gnotobiotic animals provides the gold standard for causal validation (11, 12). Methodological consensus for maternal-infant microbiota research and standardized pathways for perinatal microbiota intervention are forming (5, 193). Potential intervention strategies based on the gut-placental axis and their research status are presented (**Table 3**).

## 10 Limitations of this review

Several limitations of this review should be acknowledged. First, this is a narrative review: although sources and selection criteria are described, no formal systematic review protocol (e.g., PRISMA) was applied, and citation coverage is necessarily selective in a field producing several hundred relevant papers per year. Second, the causal spine of the gut-placental axis currently rests on a small number of animal studies, most prominently the Cell study (10), together with human association data; the strength of evidence differs by link in the chain and is flagged in the text where possible. Third, most mechanistic evidence derives from rodents, whose hemochorial placenta and short gestation differ from the human condition, so translation to human pregnancy remains uncertain. Fourth, the literature on microbial metabolites in pregnancy is evolving rapidly, and conclusions, particularly those involving 2025–2026 publications, may require revision as replication studies and clinical trials accumulate.

## 11 Summary and outlook

Intestinal signals reshape the maternal immune landscape and ultimately shape the placental immune microenvironment through three main lines: metabolite circulation, MAMP transport, and extracellular vesicle-mediated bidirectional communication. The core of this axis lies in maintaining Treg/Th17 balance, MDSC and RORγt^+^ Treg accumulation, macrophage M2-like polarization, and precise calibration of NK cell function, ensuring maternal-fetal immune tolerance and normal placental development. The study published in Cell first proved the complete causal chain of microbiota metabolites-systemic immune cells-placental tolerance within a single framework, and validated its dysregulation in human RPL cases, establishing a paradigm for the field (10).

Reviewing the evidence presented above, several general insights can be distilled. First, the intestine’s influence on the placenta is primarily through molecular signals, rather than through live bacterial colonization. The “molecular transmission” paradigm has replaced the “intrauterine colonization” hypothesis as the mainstream (23, 59). Second, effects are dose- and context-dependent: SCFAs and tryptophan derivatives tend to promote tolerance, while suprathreshold LPS and TMAO drive inflammation. Placental immune outcomes depend on the relative intensity of these two types of signals (65, 73). Third, immune cells at the maternal-fetal interface possess high metabolic plasticity, and microbiota metabolites altering cell function through metabolic reprogramming pathways is a common mechanism running through all lineages (123, 125). Fourth, causal inference tools for pregnancy microbiota research have matured, and combinations of fecal microbiota transplantation, gnotobiotic animals, Mendelian randomization, and prospective cohorts are upgrading associative findings to actionable targets (12, 132).

Under pathological conditions, microbiota dysbiosis exacerbates systemic inflammation through SCFA reduction, TMAO elevation, and LPS translocation, interacting causally with the development of PE, GDM, RPL, PTB, and FGR. Notably, obesity-related gut dysbiosis has been proposed as a shared upstream “common soil” for these complications, establishing a common pathophysiological foundation of microbial translocation, inflammatory dysregulation at the maternal-fetal interface, and abnormal placental vascularization from which GDM, PE, and FGR emerge (196). Intervention studies have shown potential for probiotics, dietary fiber, postbiotics, and exosome therapies, but causal evidence, individualized protocols, and long-term safety remain unmet challenges.

In this context, mechanistic analysis of the gut-placental immune axis suggests that when constructing humanized immune system animal models and evaluating their reproductive and developmental phenotypes, maternal gut microbiota status, microbiota metabolite profiles, and systemic immune reconstruction degrees must be incorporated into the monitoring system. When interpreting placental immune-related phenotypes, direct effects of microbiota signals should be distinguished from indirect effects of systemic immune remodeling. Pregnancy experiments in immunodeficiency backgrounds particularly require attention to standardization of microbiota variables, because the degree of immune system reconstruction itself can alter microbiota composition and its metabolic output (10, 61). In the future, the combination of single-cell multi-omics and spatial omics technologies is expected to directly resolve receptors and target cells of intestinal signals at the maternal-fetal interface in human samples, providing higher-resolution mechanistic maps for this field.
